# Single-Cell Multi-Omics Identifies Measurable Residual Disease Targets Among Myelodysplasia- and Clonal Hematopoiesis-Related Genes in Acute Myeloid Leukemia

**DOI:** 10.3390/cancers18050787

**Published:** 2026-02-28

**Authors:** Emma Frasez Sørensen, Caroline Arvé, Jonas K. Gronlund, Dorte Melsvik, Johanne Amalie Pold, Michael Knudsen, Kasper Thorsen, Anni Aggerholm, Hans Beier Ommen

**Affiliations:** 1 Department of Hematology, Aarhus University Hospital, 8200 Aarhus N, Denmark; emmase@rm.dk (E.F.S.); caarch@rm.dk (C.A.); jonago@rm.dk (J.K.G.); johpol@rm.dk (J.A.P.); anniagge@rm.dk (A.A.); 2 Department of Clinical Medicine, Aarhus University, 8200 Aarhus N, Denmark; 3 Department of Molecular Medicine, Aarhus University Hospital, 8200 Aarhus N, Denmark; michaelk@clin.au.dk (M.K.); kasper.thorsen@clin.au.dk (K.T.)

**Keywords:** acute myeloid leukemia, measurable residual disease, molecular biomarkers, single-cell multi-omics

## Abstract

Background: In acute myeloid leukemia (AML), the most sensitive measurable residual disease (MRD) methods are single-gene approaches, but these are applicable only in ~60% of AML cases. Methods: We applied multi-omics single-cell analysis on diagnostic and first remission samples to identify leukemia-specific molecular markers for subsequent MRD monitoring in six AML patients lacking AML-defining variants. Results: Five selection criteria were defined to identify suitable MRD markers. Markers of primordial leukemic clones were identified by combining data from single-cell sequencing and immunophenotyping. Specific markers suitable for use in MRD follow-up were identified in 6/6 patients, in some cases in myelodysplasia-related genes and clonal hematopoiesis-related genes usually not recommended for use in MRD determinations. Patient-specific ddPCR (limits of detection: 0.06–0.0011%) or EC-NGS assays correlated with therapeutic responses: 0/4 markers displayed molecular relapses in three non-relapsing patients, contrary to 4/4 markers of three relapsing patients. Of these, 3/4 and 1/4 markers detected molecular relapses earlier than or simultaneous with conventional methods, respectively (−115 to −338 days). Conclusions: Our results demonstrate that single-cell subclonal mapping at diagnosis and during first remission enables selection of reliable MRD targets for personalized disease surveillance in patients lacking conventional MRD markers.

## Simple Summary

Acute myeloid leukemia (AML) patients are monitored using markers reflecting residual leukemic burden during therapy and throughout post-treatment surveillance, enabling informed clinical decision-making. However, approximately 50% of AML patients lack validated molecular AML-defining variants for measurable residual disease (MRD) monitoring, due to difficulties distinguishing leukemic markers from preexisting molecular aberrations. We hypothesized that single-cell multi-omics analyses enable identification of useful MRD markers in these patients by delineating the subclonal landscape. By analyzing samples from six AML patients, we identified markers for all patients and confirmed their leukemia specificity in remission samples using droplet digital PCR (ddPCR) or error-corrected next-generation sequencing (EC-NGS), which confirmed leukemia specificity for all markers. Noteworthy, the single-cell-guided markers revealed low levels of residual disease and detected relapses earlier than conventional MRD approaches (flow cytometry and WT1 overexpression), highlighting the possibility of personalized disease monitoring for patients currently excluded from sensitive MRD assessments.

## 1. Introduction

Acute myeloid leukemia (AML) is a heterogeneous and rapidly progressing malignancy originating in the bone marrow (BM). Ultimately, the expansion of leukemic blast cells leads to BM failure and death within a few months if left untreated [1]. The prognosis for AML remains discouraging, with a three-year survival rate of ~60% for patients younger than 60 years, and ~35% for patients that are 60 years or older [2,3]. Relapse is the leading cause of mortality, contributing to the unfavorable prognosis [4]. Thus, meticulous disease surveillance assessments are recommended to closely monitor the leukemic burden [5,6].

The clinical term for such surveillance is measurable residual disease (MRD), which refers to the quantitative level of leukemic activity that remains undetectable by conventional morphological assessment. Molecular MRD detection methods include quantitative real-time polymerase chain reaction (PCR, qPCR) and digital droplet PCR (ddPCR), targeting leukemia-specific markers found in ~60% of cases [5,7,8]. Nevertheless, these single-gene approaches hold considerable potential for broader application, given that over 90% of patients present with genetic myeloid mutations at the time of diagnosis [5]. However, this would necessitate the identification of variants that can both be discriminated from preleukemic lesions and are clonally stable between diagnosis and relapse.

MRD enables evaluation of treatment response and early detection of relapse, thereby allowing for therapeutic modifications and timely intervention. Notably, MRD status following induction therapy and prior to allogeneic hematopoietic stem cell transplantation (allo-HSCT) holds prognostic value, exemplified by the association between MRD status and both overall survival and 5-year disease-free survival [6,9,10]. In recent years, MRD assessments have become increasingly integrated as a clinical decision-making tool, with emerging data demonstrating improved prognosis as a result of MRD-guided treatment modifications [11]. Further refinement of MRD surveillance strategies holds promise for improvements of AML prognosis, particularly for the patients who currently lack access to highly sensitive MRD monitoring due to the absence of conventional leukemia-specific molecular markers [5,7,8,12].

In addition to de novo AML, clonal hematopoiesis of indeterminate potential (CHIP), clonal cytopenia of undetermined significance (CCUS), and, more frequently, myelodysplastic syndrome (MDS) may ultimately progress to overt leukemia—most commonly to AML with myelodysplasia-related (MR) gene mutations [5,13]. These high-risk patients often exhibit heterogeneous clonality. From premalignant clonal hematopoietic and dysplastic subclones, additional genetic hits may drive advanced clonal outgrowth and, consequently, rapid AML transformation [14].

Mutations in genes related to clonal hematopoiesis (CH) and/or MDS are currently excluded from MRD monitoring practices, leaving a group of AML patients without AML-defining molecular markers for use in MRD [5]. Nevertheless, mutations in these genes may, in some cases, represent the actual AML-defining clone and thus serve as potential targets for MRD monitoring [15]. The challenge, however, lies in identifying such targets based on bulk material.

Single-cell multi-omics, combining genotyping and immunophenotyping, enables subclonal mapping, thereby facilitating the distinction between CH, more advanced pre-leukemic clones, and the primary AML-driving clone. Numerous studies have demonstrated the ability of single-cell multi-omics to deconvolute the clonal architecture of molecularly distinct subclones. Nevertheless, it has yet to be established whether this approach can assist in identifying molecular MRD markers in AML patients [14,16,17,18,19]. If successful, this approach may address the unmet clinical need in AML patients lacking leukemia-specific molecular markers for disease monitoring.

In this proof-of-principle study, we aimed to test if single-cell multi-omics could indeed resolve the clonal architecture of six AML cases to identify clonally stable MRD targets. We established criteria for selecting leukemia-specific MRD targets from single-cell data, based on consensus-driven insights into AML dynamics and added criteria exploiting the ability of single-cell multi-omics to accurately delineate the clonal architecture of individual leukemias. Subsequently, we retrospectively analyzed BM samples from six AML patients with MR gene mutations at two time points: at diagnosis and during first remission. Leukemia-specific MRD targets were then used for high-sensitivity ddPCR-based disease monitoring or error-corrected next-generation sequencing (EC-NGS) in longitudinal peripheral blood (PB) and BM samples. Our findings indicate that by employing this strategy, we can identify suitable MRD targets for AML patients with MR gene mutations that otherwise can only rarely be followed by molecular MRD methods.

## 2. Materials and Methods

### 2.1. Patient Samples

Eligible patients were identified based on (1) their diagnoses of AML with MR gene mutations or AML, not otherwise specified (AML-NOS) as defined by the European LeukemiaNet (ELN) since these patients do not harbor validated MRD targets [5], (2) the existence of orthogonal data from next-generation sequencing (NGS) and multiparameter flow cytometry (MFC), (3) the expression of CD34 and/or CD117 on the leukemic clone as established by MFC as necessitated to enrich for immature cell populations, (4) administration of intensive induction treatment, and (5) the availability of biobanked mononuclear cells (MNCs) from time of diagnosis and postinduction chemotherapy. The orthogonal NGS data was used to evaluate if the previously identified variants would be detectable with the DNA panel used in the present study, Table S1. We retrieved 12 BM samples collected at diagnosis and during first remission from four AML patients with MR gene mutations, one AML patient with a MR cytogenetic abnormality, and one patient with AML-NOS, all of whom were treated at the Department of Hematology, Aarhus University Hospital (AUH). The samples were collected and biobanked at the institution between 2018 and 2023 either after informed consent, or as part of the diagnostic process. In both cases, the retrieval of the samples was approved by the Central Denmark Region Committees on Health Research Ethics (record no. 1-10-72-172-23) and the Danish Data Protection Agency (record no. 1-16-02-236-24) and was conducted in accordance with the Declaration of Helsinki.

### 2.2. Routine Analyses for MRD Assessments in Clinical Practice

NGS, MFC analyses, karyotyping, and qPCR were performed for MRD assessments at the Department of Hematology, AUH, at the time of sample collection using standard clinical protocols. The targeted sequencing panel used in the clinical practice can be found in Table S1. WT1 qPCR was performed based on the assay recommended by ELN with *ABL1* and *B2M* as reference genes [20], and MRD analysis was performed as described by Skou et al. [21]. An MRD relapse was defined as a conversion from MRD negativity to MRD positivity, as recommended by the ELN guidelines [8]. Thus, a conversion from normal cytogenetics to abnormal cytogenetics, normal WT1 levels to abnormal WT1 levels, or the detection of a clone with a leukemia-associated immunophenotype (LAIP) or a blast population above the estimated normal level were defined as an MRD relapse, if confirmed in a subsequent sample. The first sample with indication of relapse was used to indicate the time of relapse detection.

### 2.3. Sample Preparation

Fresh BM aspirates were processed according to standard clinical routine procedures, Supplementary S1. Ampoules were retrieved, screw lids were briefly partially opened to relieve pressure, and ampoules were thawed by partial submersion in 37 °C water. Next, 2 mL of FCS was added dropwise to the sample and carefully mixed. Sample buffer (SB, 2% FCS [Biowest, Nuaillé, France] and 98% DPBS [Sigma-Aldrich/Merch, St. Louis, MO, USA]) was added for a total volume of 12 mL, whereafter the samples were washed and resuspended in 2 mL SB. DNase was added in a concentration of 5 mg/mL (2000 Kunits/mg) with 5 mM MgCl2, and samples were incubated for 10 min at room temperature (RT) or at 37 °C if they contained evident clumps. Cells were quantified using the automated hematology analyzer Sysmex XP-300TM (Sysmex, Kobe, Japan).

### 2.4. Identification of Germline Variants for De-Multiplexing

To allow for multiplexing of samples from different patients, germline variants were identified using the Tapestri^®^ Bulk NGS Ancillary Kit (Mission Bio, San Francisco, CA, USA) following the Tapestri^®^ Targeted Bulk DNA Sequencing protocol (MB05-0028_RevA). Details of the laboratory protocol can be found in Supplementary S2 and S3. All libraries were sequenced on a NovaSeq X Plus (Illumina, San Diego, CA, USA). Bioinformatical analyses identifying germline variants were performed using Tapestri Pipeline Merge Bulk Runs v3.4 (Mission Bio).

### 2.5. Sample Preparation and Magnetic Cell Enrichment

The single-cell laboratory procedures were performed according to the scMRD protocol developed by Mission Bio. The samples were thawed in 50% FBS, followed by quantification and viability assessment with a hematocytometer and trypan blue (Sigma-Aldrich). To ensure high viability, a “Dead Cell Removal Kit” (Stemcell Technologies, Vancouver, BC, Canada) was used. Next, a concentration of 2–5 million cells per mL was obtained with Cell Staining Buffer (CSB, BioLegend, San Diego, CA, USA). Then, cells were incubated with Human TruStain FcX (BioLegend) and Blocking Buffer (Mission Bio) for 15 min on ice. The samples were then incubated with the TotalSeq™-D scMRD AML Cocktail (BioLegend) for 30 min on ice. The cocktail included oligonucleotide-conjugated antibodies (AOC) targeting 17 cell-surface proteins frequently associated with AML, Table S3. Subsequently, immature myeloid cells were enriched using MACS^®^ Cell Separation MS Columns (Miltenyi Biotec, Bergish Glasbach, Germany), RoboSep Buffer 2 (Stemcell Technologies), CD34 MicroBeads (Miltenyi Biotec), and CD117 MicroBeads (Miltenyi Biotec). To avoid a loss of leukemic clones from cell sorting, the presence of these markers on the leukemic cells defined an eligibility criterion for the included patients, see Section 2.1. Cells positive for one or both markers were included in the enriched cell fraction. Importantly, antibody clones for CD34 and CD117 for AOC and MACS were distinct. Pre- and post-enrichment cell compositions were not evaluated for these samples.

In the case of cell aggregation, the sample was filtered using a 40 µm Flowmi™ cell strainer (SP Bel-Art, Wayne, NJ, USA). To obtain the recommended number of cells for encapsulation, the cells of each sample were quantified and viability was assessed. Samples were multiplexed for a total of approximately 150,000 cells. To ensure a sufficient number of cells, a sample with previously determined high percentage of blasts (i.e., a diagnostic AML sample) was combined with two samples previously determined to contain a low percentage of blasts (i.e., first remission samples).

### 2.6. Single-Cell DNA and Protein Sequencing

Multiplexed samples were loaded onto the Tapestri Instrument (Mission Bio) for encapsulation through microfluidics cartridges with Lysis Buffer (Mission Bio) and Reverse Primer Pool (Mission Bio) followed by cell lysis and enzymatic digestion on a thermal cycler as follows: 50 °C for 60 min, 80 °C for 10 min, and 4 °C hold. Keeping the emulsions intact, barcoding of the cell lysates was then performed on the Tapestri instrument with Barcoding Mix, Barcoding Beads, Forward Primer Pool, Antibody Tag Primer, and Barcoding Oil (Mission Bio). PCR amplification of targeted DNA regions and antibody-oligonucleotide tags was performed in a thermal cycler with program 2, Supplementary S2 and Tables S2 and S3. Next, the emulsions were broken using Extraction Agent (Mission Bio), and the PCR products were further digested with DNA Clean up Buffer (Mission Bio) and Clean up Enzyme (Mission Bio) for 60 min at 37 °C. DNA and protein PCR products were separated using AMPure XP reagent (Beckman Coulter, Brea, CA, USA) for individual cleanup with AMPure XP reagent and Streptavidin beads, respectively. Biotin Oligos (Mission Bio) were used to isolate the antibody tags of the protein PCR products. After the first cleanup of the two libraries, PCR was performed for incorporation of i5/i7 indexes (Mission Bio) with program 3, Supplementary S2. A second round of library cleanups was performed for the DNA and the protein library with AMPure XP reagent. Lastly, the libraries were quantified with a Qubit fluorometer (Invitrogen/Thermo Fisher Scientific, Waltham, MA, USA) and their purity was assessed using a 2200 TapeStation (Agilent, Santa Clara, CA, USA). The final libraries were sequenced with paired-end 150 base pair sequencing on a NovaSeq X Plus (Illumina).

### 2.7. Bioinformatic Analyses of Single-Cell Data Using Tapestri Pipeline

All bioinformatic analyses were performed using Tapestri Pipeline (Mission Bio). To enable de-multiplexing of sequenced single-cell samples, FASTQ files from bulk NGS of DNA from each of the six patients were uploaded and analyzed individually using the DNA v3.4 pipeline. Here, alignment was made to the reference genome human GRCh37/hg19, and for each patient an H5 file was generated. For each multiplexed sample, the respective H5 files from included patients were uploaded and processed using the Merge Bulk Runs v3.4 pipeline. This generated CSV files containing identified germline variants to be used for de-multiplexing. The de-multiplexing algorithm constructs a database of the germline SNPs, including the expected genotype profile for cell doublets. Each cell was assigned to the best-matching patient or cell doublet using a likelihood-based method. Cells of poor quality were removed in the following cases: If a cell was genotyped in <30% of all genotyped germline SNPs, and if the number of genotype discrepancies between the best matched patient was ≥10 or if the percentage was ≥30%.

Lastly, the FASTQ files from sequencing of the single-cell libraries were analyzed with the automated Tapestri MRD-AML DNA + Protein pipeline. In this pipeline, the CSV file for the respective multiplexed sample was also uploaded. The sequencing data was mapped onto the reference genome human GRCh37/hg19. VarSome [22] was used to annotate variants. Variant filters included the following: Depth of coverage at the given position of ≥10, genotype quality ≥ 30, variant allele frequency ≥ 35%, must be mutated in ≥3 cells, remove if synonymous, remove if near a homopolymer, remove if variants are within 300 base pairs of each other, remove if mutated in <6 cells (not applied to co-occurring variants), remove if no cells are homozygous for the variant (homozygous variants will be expected due to allelic dropouts). Germline or likely germline variants were removed. Variants were labeled as germline if either of these conditions were met: The variant was present in the gnomAD database with an allele population frequency ≥ 0.1%, or the variant was labeled as germline in the user-provided VCF file.

In multiplexed runs with panel uniformity < 80%, reported variants can be trusted, as they passed the quality filters, but the results are prone to false negatives. Thus, bulk NGS results from the clinical practice at the time of diagnosis were assessed in these cases, and any true positive variant missing in the output of the pipeline was forced through the automated pipeline if present in the data, bypassing the quality filters.

Variant allele frequencies (VAFs) were calculated from the percentage of mutated cells, taking the zygosity into account. Only cells passing the quality filters were included in the calculation of the VAFs.

### 2.8. Droplet Digital PCR

VAFs were retrospectively quantified in PB and BM samples using the QX200™ ddPCR system (Bio-Rad Laboratories Inc., Hercules, CA, USA). For each reaction well, 6–10 µL of purified DNA (approximately 200 ng of genomic DNA) was added. Samples were analyzed in duplicate or triplicate depending on assay sensitivity and sample DNA concentration. However, despite triplicate runs, DNA input occasionally remained the limiting factor rather than the limit of detection (LoD), in accordance with the rule of three (RoT) [23], particularly in highly sensitive assays or in samples with very low DNA concentrations [24]. LoD was determined during assay validation by analyzing 20 wells containing wildtype (WT) DNA:

LoD = Mean wildtype DNA + 3.08 × σ wildtype DNA (z = 3.08, α = 0.001, one-tailed)

Thus, MRD positivity (MRD(+)) was defined as a detected VAF above the LoD, or RoT in cases where DNA input was the limiting factor. In accordance with ELN guidelines, an MRD relapse was defined as a conversion from MRD negativity (VAF ≤ LoD) to MRD positivity (VAF ≥ LoD), with confirmation in a subsequent sample, or an increase in VAF of 10-fold or higher between two positive samples [8]. The first sample with indication of relapse was used to indicate the time of relapse detection.

### 2.9. Error-Corrected Next-Generation Sequencing

For patient 1, a personalized ddPCR assay could not be designed. Instead, error-corrected NGS (EC-NGS) was performed on the longitudinal samples obtained from this patient at the MOMA Core Center, Department of Molecular Medicine, Aarhus University Hospital, Denmark. Sequencing libraries (Twist Bioscience, San Francisco, CA, USA) were prepared for a panel consisting of 63 genes associated with myeloid neoplasia. The samples were sequenced on an Illumina NovaSeq 6000 at a target read depth of 10,000× using 200 ng DNA as input for library preparation and 250 ng DNA input for the hybrid capture reaction. To enable error-correction, a minimum unique alternative observation (UAO) filter of 3 was applied with fgbio (Fulcrum Genomics, Somerville, MA, USA). Variant calling was performed using a modified version of the Shearwater algorithm [25]. In the modified version, a binary approach was applied, counting reads supporting or not supporting variants while taking the strand direction into consideration. This enabled calling of complex variants such as insertions and phased variants. A threshold for variant calls was set at a maximum posterior probability of 1%. We assessed the sensitivity of variant calling by manually modifying the counts of supporting reads in each sample while maintaining the total read counts. Using this approach, the minimal number of supporting reads required for calling a variant with a posterior probability threshold of <1% was determined for each variant in each sample. Thus, for EC-NGS, MRD positivity was defined as a sample in which the variant could be confidently called using the Shearwater algorithm.

## 3. Results

### 3.1. Choosing an MRD Target: Defining Five Selection Criteria

To choose a useful MRD target among identified variants, we defined five criteria the target must comply with. To define these, we assessed the existing literature describing MRD in AML, guidelines for MRD, and the data we would obtain from our analysis. First, an ideal MRD target should be present at high levels at the time of diagnosis to indicate its association with the primordial growth of leukemic blasts instead of subclonal emergence [26]. Leukemic blasts are, by definition, present at minimum 20% for AML but can constitute more than 90% of cells in the blood at diagnosis [5]. Thus, a minimum VAF of 10%, corresponding to one mutated allele in 20% blasts, is required for the MRD target. Second, pre-leukemic mutations have been reported to persist during remission, while leukemia-specific mutations correlate with therapeutic responses [27,28,29,30]. Thus, when patients are treated with the first course of intensive induction therapy, it is expected that leukemic blasts and their associated genetic variants decrease to levels that are below the detection limits of the analysis. Third, leukemic cells are characterized by an immature immunophenotype, and one or more LAIPs are often present in a patient. The LAIPs are characterized by asynchronous antigen expression, i.e., expression of markers from different developmental stages in a lineage, and cross-lineage marker expression, e.g., expression of markers from B cells or T cells [31,32]. Using single-cell multi-omics data, it is possible to determine the immunophenotypes of dominating clones as well as subclones. Fourth, clonal selection can pressure leukemic cells to lose genetic variants over time, which could lead to false negative MRD assessments in the case where a subclone is not monitored with the chosen target [14,33]. With single-cell multi-omics data, the clonality of a patient’s leukemia is revealed, thereby enabling assessment of subclonal development. Lastly, the ideal biomarker is conserved throughout the disease course [27]. For example, mutations in *RAS* and *FLT3* have previously been reported to disappear at relapse.

The criteria were defined as follows:

1. The target is present in a large clone at diagnosis (target VAF ≥ 10% across clones);
2. The target is reduced to <1% VAF after the first treatment course;
3. The target is present in a clone with a distinct, immature immunophenotype that displays aberrant characteristics (i.e., an immunophenotype with unusual combinations of surface markers);
4. The target is not missing or lost in any clone at a more advanced stage (e.g., due to loss of heterozygosity, LOH);
5. The target is not known to occasionally become negative at relapse [27].

Aberrant immunophenotypes were detected using a different from normal (DfN) approach integrated with a diagnostic LAIP [5,6].

### 3.2. Clinical Characteristics and Sample Availability

Six patients diagnosed with AML with MR gene mutations (n = 4), AML with MR cytogenetic abnormalities (n = 1), or AML, NOS (n = 1) between 2018 and 2023 were included in the study, Table 1 and Table S4. Men and women were equally represented, and the mean age at diagnosis was 56 years (range 42–66). Five patients underwent myeloablative (n = 1) or non-myeloablative (n = 4) allo-HSCT during the study follow-up period and three patients experienced disease relapse.

MFC analysis from the clinical practice confirmed an LAIP of either CD34^+^, CD117^+^, or CD34^+^/CD117^+^, thereby providing a rationale for sorting these immature cell populations. For all patients, BM-derived MNCs obtained at the time of diagnosis and during first remission were retrospectively sorted and analyzed using single-cell multi-omics. The mean interval between the samples was 44 days (range 30–61).

### 3.3. Single-Cell Multi-Omics Identifies Leukemia-Specific MR Targets

**Figure 1:**
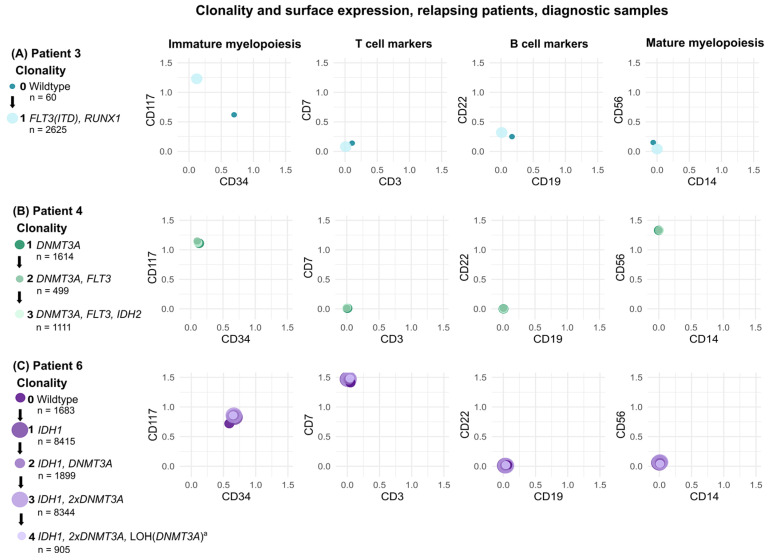
Clonality and immunophenotype by single-cell multi-omics of cells derived from diagnostic samples of relapsing patients. Clones are depicted as spheres, color-coded by developmental stage, and scaled according to number of cells belonging to the respective clone. (**A**–**C**) Left panels depict the detected clones, the developmental stages, and associated genotypes of patients 3, 4, and 6, respectively. Right panels depict the immunophenotypes of each clone in 2D plots of specified surface markers. Immunophenotypes are based on centered log-transformed (CLR)-values of surface antigen expression, which can be found in Table S7. ^a^ = Loss of heterozygosity (LOH) affected both *DNMT3A* variants oppositely. *DNMT3A* p.F640* was lost with LOH, while the wildtype allele at position 729 was lost, leaving only the *DNMT3A* p.R729G variant at this position.

**Figure 2:**
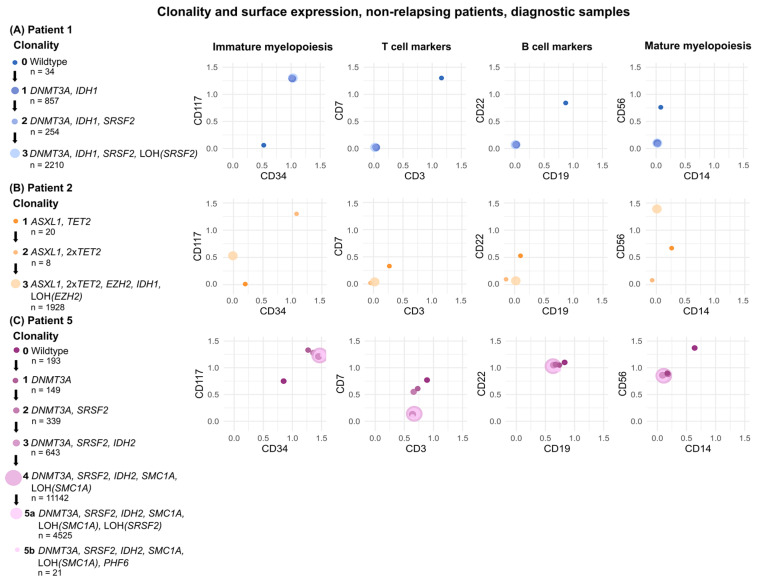
Clonality and immunophenotype by single-cell multi-omics of cells derived from diagnostic samples of non-relapsing patients. Clones are depicted as spheres, color-coded by developmental stage, and scaled according to number of cells belonging to the respective clone. (**A**–**C**) Left panels depict the detected clones, the developmental stages, and associated genotypes of patients 1, 2, and 5, respectively. Right panels depict the immunophenotypes of each clone in 2D plots of specified surface markers. Immunophenotypes are based on CLR-values of surface antigen expression, which can be found in Table S7.

A total of 56,067 cells were analyzed across all 12 samples, of which 49,479 cells were derived from diagnostic samples and 6588 cells were derived from first remission samples. The number of cells analyzed per sample ranged from 1965 to 21,246 cells (mean 8247 cells) in diagnostic samples and 357 to 1710 cells (mean 1098) in remission samples, Figure 1 and Figure 2 and Figures S1 and S2. Across diagnostic samples, 21 variants were detected in a total of 11 genes, Figure 1 and Figure 2. The number of identified subclones varied both among patients and between time of sampling. At diagnosis, a mean of three subclones (range one-six) were detected across patients. In comparison, the mean number of subclones detected during first remission was one (range zero-five, *p* = 0.086). For each processed run, quality parameters were obtained, revealing a panel uniformity < 80% in three runs, a varying number of cells analyzed per run (range 7287–23,013 cells), Table S5.

Based on multi-omics data distinguishing immunophenotypically and genomically distinct subclones, targets fulfilling the defined selection criteria for leukemia specificity were identified in all cases, Table 2, Tables S4 and S6. In two cases, two targets met these criteria, Table S6. Thus, for all patients one (n = 4 patients) or two (n = 2 patients) targets were selected for subsequent sensitive ddPCR VAF quantification (limit of detections (LoDs) 0.06–0.0011%), Table 2. None of the identified targets are validated for MRD assessment in current clinical practice [5,6,8].

### 3.4. Detected Subclones Exhibit Multiple Immunophenotypes

For all detected subclones, a common immunophenotype was determined based on the mean CLR values of antigen expression for the specific clone, Figure 1 and Figure 2 and Tables S7 and S8. However, when inspecting individual cells of each subclone, it was evident that most subclones contained cells with varying surface expressions. For example, in the diagnostic sample of patient 4, three clones were detected, Figure 1B. These clones shared a similar immunophenotype of CD117^+^/CD34^−^ in accordance with the LAIP determined by routine diagnostics. Nonetheless, some of the cells in each of the clones appeared to be CD117^+^/CD34^+^, Figure S3A. Similarly, clones in the diagnostic sample of patient 5 were predominantly CD117^+^/CD34^+^, in agreement with the MFC-determined LAIP, though a small population of cells were CD117^−^/CD34^+^, Figure S3B. Interestingly, the LAIP determined by routine diagnostics for patient 6 comprised two populations: One CD117^+^/CD34^+^ and another CD117^+^/CD34^−^. From the single-cell analysis, it was clear that both immunophenotypes as well as some CD117^−^ cells were present across clones, Figure S3C.

These findings indicate that single-cell-based identification of leukemia-specific MRD targets should primarily rely on genotypic clonality. Nevertheless, immunophenotypic information remains a critical parameter in supporting the genotypic findings, consistent with our proposed criteria for target selection.

### 3.5. Single-Cell-Guided Targets Detect Early Molecular Relapses and Deficient Therapy Response

We compared the ability of the single-cell-guided targets to detect MRD with the conventional MRD approaches in the routine laboratory, implemented in adherence to the ELN MRD guidelines. Sample availability for subsequent ddPCR analysis of the single-cell-guided targets differed among the patients, Table S9. A median of six PB samples and six BM samples were analyzed using ddPCR (ranges 4–29 and 4–11, respectively).

**Figure 3:**
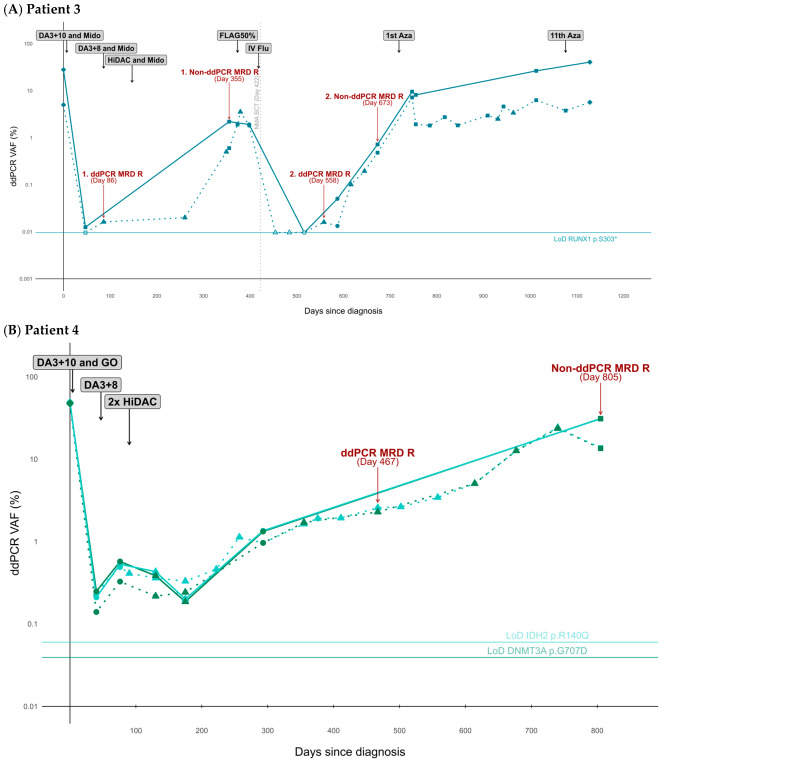

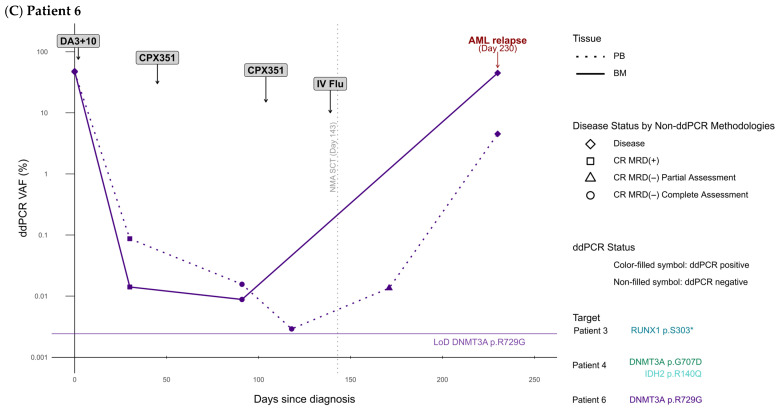
Highly sensitive ddPCR for disease surveillance targeting single-cell-guided MRD markers for relapsing AML. Three relapsing AML patients without validated MRD markers were monitored by quantification of VAFs using (**A**) *RUNX1* S303*, (**B**) *DNMT3A* p.G707D and *IDH2* p.R140Q, and (**C**) *DNMT3A* p.R729G. PB and BM assessments are depicted using dotted and solid lines, respectively. Each data point is formatted according to the conventionally determined MRD status as defined in clinical practice, adhering to the ELN guidelines, Table S10. CR MRD(−) Partial Assessment is defined as MRD(−) across all applied MRD technologies when only a subset of the available technologies was analyzed. The target LoD thresholds are represented as horizontal lines. Negative samples (ddPCR VAF < LoD) are non-filled, whereas positive samples are color-filled (ddPCR VAF > LoD). Abbreviations: Azacitidin, Aza; bone marrow, BM; liposomal cytarabine and daunorubicin, CPX351; complete remission, CR; daunorubicin and cytarabine, DA; digital droplet PCR, ddPCR; European LeukemiaNet, ELN; Fludarabine, arabinofuranosyl cytidine, and granulocyte colony-stimulating factor, FLAG; Gemtuzumab, ozogamicin, GO; high-dose cytarabine, HiDAC; intravenous fludarabine, IV flu; limit of detection, LoD; midostaurin, Mido; measurable residual disease, MRD; MRD-relapse, MRD R; non-myeloablative stem cell transplantation, NMA SCT; peripheral blood, PB; variant allele frequency, VAF.

Interestingly, in two of the three patients with relapsed AML, ddPCR targeting patient-specific markers detected relapses earlier than conventional MRD assessment methods (patient 3 = −269 days at day +86 vs. day +355 and again −115 days at day +558 vs. day +673, patient 4 = −338 days at day +467 vs. day +805, where day = 0 is the day of diagnosis), Figure 3, Table 3, and Tables S13 and S14. In the remaining relapsing AML case, patient 6, the criteria for MRD relapse did not align with the ELN definitions, despite an increasing PB VAF on day +171, e.g., 28 days post-HSCT (from 0.0029% prior to allo-HSCT to 0.0135% post-allo-HSCT), Figure 3C and Table S16. Notably, prospective application of this single-cell-guided ddPCR MR3D approach would likely have enabled the detection of an early molecular MRD relapse in patient 6. The increasing VAF would have warranted a prompt confirmatory assessment to ascertain the presence of an emerging relapse. Importantly, the complex cytogenetic changes in patient 6 were detected at diagnosis and at the time of relapse on day +230, but not at any other times.

Next, we assessed the ability of single-cell-guided ddPCR targets to evaluate therapy response. Following the first course of induction therapy, all relapsing patients received additional intensive chemotherapy. In all these cases, remission samples demonstrated CR MRD(−) as assessed by conventional non-ddPCR MRD approaches, whereas ddPCR MRD analysis indicated MRD positivity in relapsing patients, underscoring the superior sensitivity of molecular ddPCR MRD measurements, Figure 3, Table 3, and Tables S13, S14 and S16. Of great importance, we detected continuous MRD positivity in PB samples derived from patient 3 on days 379 and 398 (VAF = 3.54% and 1.82%), as well as in a BM sample from day 398 (VAF = 1.91%), Figure 3A and Table S13. This patient was monitored using WT1 overexpression, which had detected a molecular relapse on day +355, resulting in the administration of FLAG treatment. Contradictory to the subsequent MRD assessment by WT1 overexpression, single-cell-guided ddPCR revealed a sustained leukemic burden pointing to an ineffective treatment prior to allo-HSCT. Importantly, immediately prior to and post-allo-HSCT, the non-ddPCR methodologies established the disease status for the relapsing patients as complete remission (CR) MRD(−), contradictory to our findings.

### 3.6. DNMT3A Mutations as Leukemia-Specific MRD Targets

We hypothesized that for some patients, variants in *DNMT3A* may be confined to the leukemia-driving AML clone, despite literature demonstrating pre-leukemic involvement [5]. Single-cell-guided targets in *DNMT3A* were identified for two patients (patient 4 and 6), Table 2 and Figure 3B,C. In both cases, a decrease in VAF was observed after the first course of DA3 + 10 (patient 4, BM VAF 48.26% to 0.25%; patient 6, BM VAF 48.09% to 0.01%), Tables S14 and S16. A response to therapy was also observed after subsequent intensive chemotherapy for both patients. For patient 4, it was evident that secondary intensive chemotherapy had stabilized the leukemia. The observed changes in VAFs in response to first induction treatment indicate leukemia specificity of the selected single-cell-guided *DNMT3A* targets.

MRD often manifests as a very low leukemia burden, thereby underscoring the importance of detection methods capable of deep quantification. The LoDs of the two *DNTM3A* assays are 0.039 (p.G707D) and 0.0024 (p.R729G), with the latter being surpassed only by the highly sensitive *EZH2* assay, Table 2. Differences in LoDs are due to variations in target sequence properties, which to varying degrees enables the design of a sensitive assay. Compared to the remaining five assays, the *DNMT3A* assays exhibited greater sensitivity than the *IDH1* and *IDH2* assays. Thus, in patient 4, the *DNMT3A* assay would have been preferred in a single gene ddPCR MRD strategy, even though *IDH2* demonstrates similar leukemia specificity. Notably, patient 1 had a *DNMT3A* variant that was not identified as representing the leukemia-driving clone, thus highlighting the advantages of target selection based on single-cell multi-omics data, which allows for distinguishment between pre-leukemic and leukemic variants within the same gene.

These results indicate that single-cell multi-omics enables the identification of leukemia-specific, personalized targets for subsequent high-sensitivity ddPCR MRD surveillance, utilizing targets that are currently excluded from the MRD practice due to pre-leukemic implications.

### 3.7. Single-Cell-Guided MRD Targets Remain Negative in Sustained Remission

For patients with non-relapsing AML (patients 1, 2, and 5) leukemia-specific variants were present in *SRSF2* (p.P95H), *EZH2* (p.T592*) and *IDH1* (p.R132C), and *IDH2* (p.R140Q), respectively, Figure S4A–C, and Tables S11, S12 and S15. These targets confirmed continuous low levels of the disease burden. As it was not possible to design a ddPCR assay for the single-cell-guided target in *SRSF2* found in patient 1, the variant was tracked using targeted NGS. This enabled parallel detection of the remaining variants in *DNMT3A* and *IDH1*, not predicted to be leukemia-specific, Table 2. Of these variants, only the *SRSF2* remained MRD negative, correlating with the therapeutic response and clinical data of the patient, Figure S4A. In patient 2, both selected markers remained negative by ddPCR until the end of follow-up at day +489, with *EZH2* becoming negative 167 days later than *IDH1*, possibly owing to the higher sensitivity of the *EZH2* assay. In patient 5, a small increase in the VAF of *IDH2* (p.R140Q) in BM was observed on day +211, i.e., 83 days post-allo-HSCT (MRD(−) VAF 0.06% to MRD(+) VAF 0.07%) and on day +491, i.e., 363 days post-allo-HSCT (MRD(−) VAF 0.06% to MRD(+) VAF 0.07%), Figure S4B,C and Table S15. Thus, this could represent residual leukemia held in check by the Graft-versus-leukemia effect as earlier reported [34].

## 4. Discussion

Approximately half of all AML cases are currently excluded from molecular disease surveillance, primarily due to the risk of tracing premalignant cells rather than overt leukemia. To overcome this, we employed single-cell multi-omics to identify leukemia-specific MRD targets in six AML patients lacking conventional AML-defining variants. Single-cell-guided ddPCR identified incomplete treatment responses in patients previously considered well-treated based on conventional non-ddPCR MRD assessments. Essentially, 0/4 single-cell-guided targets displayed molecular relapses in non-relapsing patients, compared to 4/4 in the three relapsing patients. Of note, 3/4 targets in the relapsing patients detected an early ddPCR MRD relapse, with the remaining target potentially showing similar results with more frequent sampling. While the cohort was limited to six patients, it comprised a homogenous group of AML patients, five of whom had AML with MR gene mutations, and one with AML-NOS who had a gene mutation in SETBP1, a gene frequently mutated in MDS [35].

Integrating current consensus knowledge of AML clonal dynamics during treatment and relapse with the capacity of single-cell multi-omics to resolve the AML subclonal landscape, we established five criteria for MRD target selection. In 6/6 patients, AML-specific MRD targets were identified based on variants fulfilling these criteria. Notably, the two criteria that relied solely on single-cell multi-omics—criterion #3 (the target must be present in a distinct, immature, and aberrantly expressed immunophenotype) and criterion #4 (the target must not be lost in any more advanced subclone)—proved most critical, leading to exclusion of three and five variants, respectively, across the six patients. Only one other criterion, criterion #2 (the target VAF < 1% following the first induction course), proved essential, resulting in six variant rejections. Hitherto, based on bulk analyses, consensus recommendations regarding targets in molecular MRD monitoring have been difficult to deliver [6]. The present observations provide some explanation for this.

Our selected MRD targets exhibited AML specificity across all patients, as evidenced by their complete or near-complete elimination following intensive chemotherapy, rising levels in relapsing patients, and sustained negativity in non-relapsing patients, as shown by the retrospective ddPCR analyses. Moreover, targeted NGS results revealed that the variants which did not fulfill the selection criteria remained positive until HSCT, underscoring their lack of suitability as MRD targets as opposed to the selected target, further demonstrating the effectiveness of the single-cell-guided target selection approach. Such leukemia-specific molecular disease surveillance may inform clinical decision-making, including the identification of patients who may benefit from treatment modification and early intervention upon relapse—both applications are particularly relevant given the poor chemotherapeutic responses and high relapse risk associated with AML in older adults [36].

Of the patients included in this study, prospective MRD monitoring could have contributed to improved AML management. First, detection of molecular relapses could have enabled treatment initiation 269 and 115 days earlier in patient 3, and 338 days earlier in patient 4, respectively. Second, clinicians might have considered treatment intensification with FLAG-IDA for all relapsing patients, prompted by ddPCR-identified persistent leukemic cells following induction therapy—consistent with improved outcomes from therapy escalation in patients with residual disease, as evidenced by the UK MRC AML18 study [37]. Moreover, patients 3 and 4 were not transplanted in first remission due to comorbidities making allogeneic transplants high-risk procedures. Knowledge of MRD status prior to the transplant decision in these patients could have qualified this choice. Finally, an inadequate response to re-induction therapy (FLAG50%) during management of the patient 3 first relapse (day +355) would have been identified—specifically, a molecular leukemic stagnation in contrast to conventional MRD approaches (morphology, MFC, and *WT1* overexpression), which falsely indicated CR.

Previous studies have found that *DNMT3A*, *TET2*, and *ASXL1* (DTA) and certain MR gene mutations are more common in CH than mutations in *IDH* genes, suggesting a stronger link between *IDH* mutations and rapid AML progression [38,39]. Notably, *IDH1* mutations tend to present with lower VAFs at diagnosis and are more frequently cleared in CR compared to *IDH2* mutations—possibly reflecting even greater leukemia specificity [40]. In our study, the two single-cell-guided *DNMT3A* variants (patients 4 and 6) and the *EZH2* mutation (patient 2) exhibited leukemia specificity comparable to that of co-occurring *IDH* mutations. These non-*IDH* targets were preferred as MRD markers due to their ability to detect extremely low levels of leukemic burden. Importantly, *IDH* genes have previously demonstrated confined assay sensitivity compared to other genes [41,42]. Accordingly, we introduced a target selection criterion that prioritized assay sensitivity over literature-based assumptions of increased AML specificity of *IDH* mutations, as single-cell multi-omics enabled case-by-case evaluation of this specificity in individual patients.

A key limitation of the study is the inclusion of only six AML cases. In addition, ddPCR MRD was quantified retrospectively, limiting our data points to those time points at which patients had PB and BM samples collected, and surplus material stored in the laboratory biobank. Accordingly, prompt confirmatory testing in accordance with the ELN guidelines was not performed in a standardized manner [6]. Thus, validation of our findings in prospective, larger cohorts remains warranted.

In a recent randomized, controlled phase 3 trial by Potter et al., regular molecular MRD monitoring in an overall AML patient cohort did not confer a survival benefit compared to patients who did not undergo MRD assessment [11]. In contrast, in a pre-specified AML subgroup analysis comprising patients with *NPM1* and co-mutated *FLT3* internal tandem duplication (ITD), routine MRD surveillance was associated with a significant improvement in prognosis. This discrepancy may, in part, be attributable to subgroup-specific differences in disease aggressiveness and treatment options. Low-risk patients relapse less frequently, and in cases of full AML relapse, a second remission is often achievable, reducing the clinical benefit of MRD follow-up. In contrast, intermediate-risk *NPM1*+*FLT*-*ITD*+ AML is associated with elevated relapse risk and poorer re-induction response, underscoring the value of MRD surveillance for early molecular disease control before refractory, often fatal, AML develops.

As well as the *NPM1*+*FLT*-ITD+ AML subgroup, AML with MR gene mutations exhibit a high-risk prognosis with high prevalence of relapse and/or refractory disease. Moreover, our single-cell-identified MRD targets provide a specificity equal to that of *NPM1* with good and, in some cases, excellent sensitivity (LoD range: 0.001–0.06%), albeit in few samples. Accordingly, it appears reasonable to propose the potential for achieving a prognostic improvement similar to that observed in the aforementioned subgroup, in the hypothetical scenario of implementing our combined single-cell multi-omics and ddPCR-based MRD strategy—or, more plausibly, an optimized future version incorporating refined target selection criteria and methodological improvements to single-cell multi-omics.

A further limitation of our study was the constrained coverage of the single-cell gene panel, which did not include certain myeloid hotspot variants. Specifically, bulk-identified mutations in *SETBP1* (patient 6) and *DNMT3A* (patient 3) were not captured, while the single-cell-identified *SMC1A* mutation (patient 5) was absent from the bulk panel. In patient 4, a *PTPN11* variant was not detected despite being included in the single-cell gene panel, possibly due to loss during CD117^+^ and/or CD34^+^ enrichment, or due to limitations in single-cell multi-omics sensitivity. The former may reflect findings by Struyf et al., who reported CD117 downregulation following cryopreservation [43]. Accordingly, future studies should consider immediate gating and broader myeloid gene panel coverage to better resolve the subclonal landscape.

As shown by Dillon et al., MRD remains an imperfect and primarily prognostic marker, with ~30% of MRD(+) AML patients not experiencing relapse [44]. Following induction therapy, leukemic burden was evaluated using a 29-gene panel, from which DTA mutations were excluded in the subsequent MRD positivity assessments. Consequently, MR mutations may still reflect CH, while AML-specific DTA variants in some patients were excluded. Supporting the rationale of Dillon et al. for excluding DTA mutations in MRD contexts, a study by Jongen-Lavrencic et al. found that DTA persistence in CR did not correlate with increased relapse rates, indicating that these mutations lack MRD potential [29].

In contrast, our study demonstrates that single-cell multi-omics might resolve the confounding effects of CH- and MR-related clonality while simultaneously identifying AML-specific targets within the same mutational categories. Although a precise single-cell-guided MRD approach does not yet translate into standardized treatment algorithms, the accuracy achieved here marks a step toward individualized, molecularly informed AML management.

## 5. Conclusions

In conclusion, in this proof-of-principle study we demonstrate that by understanding subclonal architecture in individual patient leukemic clones, leukemia-specific MRD targets can be identified even for AML subtypes characterized by presence of mutations often associated with myelodysplastic neoplasia or clonal hematopoiesis, such as AML, myelodysplasia-related (AML-MR) or AML-NOS. The potential possibility for molecular disease surveillance in AML-MR and AML-NOS could allow for clinical decision-making on a better informed basis, plausibly improving the otherwise poor prognosis associated with these diseases. Of course, the limited number of patients in the present study necessitates either larger studies using single-cell technologies or the application of the insights gained in this study to develop the selection of markers in AML-MR and AML-NOS patients using NGS or ddPCR-based technologies.

## Figures and Tables

**Table 1:** Clinical characteristics of patients.

	Patients n (% or Range)
Gender	
Male	3 (50)
Female	3 (50)
Age at AML diagnosis, mean (range)	56 (42–66)
Previous MDS diagnosis	0 (0)
BM blast percentage at diagnosis, mean (range)	67% (30–91%)
Cytogenetic aberrations at diagnosis	2 (33)
Relapse	3 (50)
Death	2 (33)
Transplantation	5 (83)
Myeloablative	1 (33)
Non-myeloablative	4 (67)
First course of induction therapy	
DA3 + 10	5 (83)
CPX-351	1 (17)
Second course of induction therapy	
DA3 + 8	3 (50)
CPX-351	1 (17)
FLAG-Ida	1 (17)
None	1 (17)
Additional chemotherapy	
HiDAC	3 (50)
Azacitidine	1 (17)
FLAG	1 (17)
None	1 (17)

Abbreviations: Acute myeloid leukemia, AML; bone marrow, BM; liposomal cytarabine and daunorubicin, CPX-351; daunorubicin and cytarabine, DA; fludarabine, arabinofuranosyl cytidine, granulocyte colony-stimulating factor, FLAG; idarubucin, Ida; myelodysplastic syndrome, MDS.

**Table 2:** Genetic variants identified by clinical routine NGS and single-cell-guided targets for each patient.

Patient	Bulk NGS Variants (VAF%)	Selected Target(s)	Assay Sensitivity (LoD (%))
1	*SRSF2* p.P95H (46) *DNMT3A* p.P904L (45) *IDH1* p.R132C (47)	*SRSF2* (p.P95H)	0.046–0.059 0.18–0.21 0.19–0.21
2	*ASXL1* p.E635Rfs*15 (47) *EZH2* p.T592Vfs*82 (93) *IDH1* p.R132C (42) *TET2* p.I1139Lfs*13 (47) *TET2* p.N1387S (48)	*EZH2* (p.T592*), *IDH1* (p.R132C)	0.0011, 0.059
3	*DNMT3A* p.? c.1555-2A>G (42) *FLT3*(ITD) p.E611_F612ins17 (31) *RUNX1* p.S303* (31)	*RUNX1* (p.S303*)	0.0097
4	*DNMT3A* p.G707D (47) *FLT3* p.V592G (45) *FLT3*(TKD) p.N841I (3) *IDH2* p.R140Q (49) *PTPN11* p.S502L (3)	*DNMT3A* (p.G707D), *IDH2* (p.R140Q)	0.039, 0.06
5	*DNMT3A* p.R882C (39) *IDH2* p.R140Q (25) *SRSF2* p.P95H (40)	*IDH2* (p.R140Q)	0.06
6	*DNMT3A* p.F640* (49) *DNMT3A* p.R729G (48) *IDH1* p.R132G (48) *SETBP1* p.T1078M (47)	*DNMT3A* (p.R729G)	0.00242

Abbreviations: Limit of detection, LoD; Next-generation sequencing, NGS.

**Table 3:** Comparison of the earliest time point of MRD positivity stratified by the detection method.

First Day of MRD Positive Sample Detected in Relapsed Patients by Method										
	ddPCR		MFC		WT1		Cytogenetics		Morphology	
	Day	Testing interval	Day	Testing interval	Day	Testing interval	Day	Testing interval	Day	Testing interval
Patient 3	86, 558	30	N/A	84	355, 673	30	N/A	N/A	N/A	100
Patient 4	467	45	805	108	805	45	N/A	N/A	N/A	217
Patient 6	230	56	230	56	N/A	N/A	230	61	230	130

Day numbers indicate days after diagnosis. Testing interval is given as the median number of days between two consecutive analyses in PB or BM. Not applicable (N/A) is indicated if analysis was not performed for the patient or if the analysis did not detect MRD positivity at any of the analyzed time points.

## Data Availability

Data included in the current study are available upon reasonable request.

## Supplementary Materials

The following supporting information can be downloaded at: https://www.mdpi.com/article/10.3390/cancers18050787/s1, Supplementary S1: Harvesting and cryopreservation of mononuclear cells; Supplementary S2: PCR programs for single cell DNA and protein sequencing; Supplementary S3: Targeted bulk DNA sequencing for demultiplexing; Table S1: DNA sequencing panel used in the clinical practice routine analyses; Table S2: DNA panel for single-cell multi-omics sequencing; Table S3: Antibody-oligonucleotide conjugate panel for single-cell multi-omics sequencing; Table S4: Genetic characteristics of patients determined by routine sequencing analyses at diagnosis; Figure S1: Deconvolution of clonality in first remission samples of relapsing patients; Figure S2: Deconvolution of clonality in first remission samples of non-relapsing patients; Table S5: QC values of multiplexed single-cell multi-omics samples; Table S6: Variants excluded using the five selection criteria (1–5) for target selection; Table S7: Antigen expression of clones (>1) and wildtype cells (0) per diagnostic patient sample by single-cell multi-omics; Table S8: Antigen expression of clones (>1) and wildtype cells (0) per first remission patient sample by single-cell multi-omics; Figure S3: CD34 expression (x-axis) vs. CD117 expression (y-axis) of cells in the diagnostic samples of patients 4–6; Table S9: Sample availability for ddPCR analyses; Table S10: Description of symbols indicating disease status by non-ddPCR methodologies; Table S11: Patient 1, table with VAFs quantified using targeted sequencing supporting Figure S4A; Table S12: Patient 2, table with ddPCR quantified VAFs supporting Figure S4B; Table S13: Patient 3, table with ddPCR quantified VAFs supporting Figure 3A; Table S14: Patient 4, table with ddPCR quantified VAFs supporting Figure 3B; Table S15: Patient 5, table with ddPCR quantified VAFs supporting Figure S4C; Table S16: Patient 6, table with ddPCR quantified VAFs supporting Figure 3C; Figure S4: Highly sensitive ddPCR and EC-NGS for disease surveillance targeting single cell-guided MRD markers for non-relapsing AML.
