# Supplementary material for: Single-Cell Multi-Omics Identifies Measurable Residual Disease Targets Among Myelodysplasia- and Clonal Hematopoiesis-Related Genes in Acute Myeloid Leukemia
Source: Cancers (Basel). 2026 Feb 28;18(5):787. doi: 10.3390/cancers18050787 (PMC12984621; doi:10.3390/cancers18050787)

# Supplementary methods, tables and figures for *Single-cell multi-omics identifies measurable residual disease targets among myelodysplasia- and clonal hematopoiesis-related genes in acute myeloid leukemia*

**Supplementary S1: Harvesting and cryopreservation of mononuclear cells**

Using density gradient centrifugation on LymphoPrep Stemcell Technologies, Vancouver, Canada), mononuclear cells (MNCs) were harvested from PB and BM samples. Erythrocytes were removed using lysis buffer (Ampliqon, Odense, Denmark). The MNCs were cryopreserved in liquid nitrogen tanks at -165°C in a storage solution of 10% DMSO (Sigma-Aldrich/Merch, St. Louis, MO, USA), 50% fetal calf serum (FCS) (Biowest, Nuaillé, France), and 40% RPMI (Invitrogen/Thermo Fisher Scientific, Waltham, MA, USA).

**Supplementary S2: PCR programs for single cell DNA and protein sequencing**

Program 1 (PCR amplification, bulk NGS): 98°C for 6 minutes (4°C/second); 10 cycles of 95°C for 30 seconds, 72°C for 10 seconds, 61°C for 9 minutes, 72°C for 20 seconds (1°C/second); 10 cycles of 95°C for 30 seconds, 72°C for 10 seconds, 48°C for 9 minutes, 72°C for 20 seconds (1°C/second); 72°C for 2 minutes (4°C/second); and 4°C hold overnight.

Program 2 (PCR amplification): 98°C for 6 minutes (4°C/second); 11 cycles of 95°C for 30 seconds, 72°C for 10 seconds, 61°C for 9 minutes, 72°C for 20 seconds (1°C/second); 13 cycles of 95°C for 30 seconds, 72°C for 10 seconds, 48°C for 9 minutes, 72°C for 20 seconds (1°C/second); 72°C for 2 minutes (4°C/second); and 4°C hold overnight.

Program 3 (Library preparation): 95°C for 3 minutes; 10 cycles (DNA libraries) or 20 cycles (protein libraries) of 98°C for 20 seconds, 62°C for 20 seconds, 72°C for 45 seconds; 72°C for 2 minutes; and 4°C hold

**Supplementary S3: Targeted bulk DNA sequencing for demultiplexing**

Following the Tapestri® Targeted Bulk DNA Sequencing protocol (MB05-0028_RevA), the concentrations of previously purified DNA from MNCs available from any sample of each patient

were measured on a NanoDrop 2000. All DNA products were within the required range of 5-50 ng/uL (range 11.3-37.7 ng/uL). Primers were added in a 1/10 dilution and targeted PCR amplification was performed using program 1, SM2. Next, the PCR products were digested on a thermal cycler at 37°C

for 60 minutes. The targeted PCR products were cleaned up using AMPure XP reagent (Beckman Coulter, Brea, CA, USA, cat.: A63881), followed by a library PCR amplification step for ligation of library indexes: Program 3, SM2. Amplified PCR products were cleaned up using AMPure XP reagent (Beckman Coulter), and libraries were quantified on a Qubit Fluorometer (Invitrogen) and on a TapeStation (Agilent, Santa Clara, CA, USA) using the high sensitivity D1000 assay (Agilent) at Department of Molecular Medicine, AUH.

**Table S1: DNA sequencing panel used in the clinical practice routine analyses.** The panel includes 30 genes.

| **Gene** | **Coding sequence (CDS)/ hotspot (HS)** | **Gene** | **Coding sequence (CDS)/ hotspot (HS)** |
|---|---|---|---|
| *ABL1* | HS | *KIT* | HS |
| *ASXL1* | HS | *KRAS* | HS |
| *BRAF* | HS | *MPL* | HS |
| *CALR* | HS | *NPM1* | HS |
| *CBL* | HS | *NRAS* | HS |
| *CEBPA* | CDS | *PTPN11* | HS |
| *CSF3R* | CDS | *RUNX1* | CDS |
| *DNMT3A* | CDS | *SETBP1* | HS |
| *ETV6* | CDS | *SF3B1* | HS |
| *EZH2* | CDS | *SRSF2* | HS |
| *FLT3* | HS | *TET2* | CDS |
| *HRAS* | HS | *TP53* | CDS |
| *IDH1* | HS | *U2AF1* | HS |
| *IDH2* | HS | *WT1* | HS |
| *JAK2* | CDS | *ZRSR2* | CDS |

**Table S2: DNA panel for single-cell multi-omics sequencing.** This Tapestri scMRD AML DNA Panel was a catalogue panel from MissionBio. For further details on the panel construction, we refer to Mission Bio's ressources. The panel included 43 genes, seven of which were used for sample demultiplexing.

| Gene | Objective | Gene | Objective |
|---|---|---|---|
| *ASXL1* | Variant detection | *NPM1* | Variant detection |
| *BCOR* | Variant detection | *NRAS* | Variant detection |
| *BRAF* | Variant detection | *PHF6* | Variant detection |
| *CALR* | Variant detection | *PPM1D* | Variant detection |
| *CBFB* | Variant detection | *PTPN11* | Variant detection |
| *CBL* | Variant detection | *RAD21* | Variant detection |
| *CEBPA* | Variant detection | *RUNX1* | Variant detection |
| *CHEK2* | Variant detection | *SETBP1* | Variant detection |
| *CSF1R* | Variant detection | *SF3A1* | Demultiplexing |
| *CYP4F3* | Demultiplexing | *SF3B1* | Variant detection |
| *DNMT3A* | Variant detection | *SMC1A* | Variant detection |
| *ETV6* | Variant detection | *SRSF2* | Variant detection |
| *EZH2* | Variant detection | *STAG2* | Variant detection |
| *FLT3* | Variant detection | *TET2* | Variant detection |
| *GATA2* | Variant detection | *TP53* | Variant detection |
| *IDH1* | Variant detection | *TRPC4* | Demultiplexing |
| *IDH2* | Variant detection | *U2AF1* | Variant detection |
| *IL6R* | Demultiplexing | *UBA1* | Demultiplexing |
| *IP6K1* | Demultiplexing | *WT1* | Variant detection |
| *JAK2* | Variant detection | *ZEB2* | Demultiplexing |
| *MYC* | Variant detection | *ZRSR2* | Variant detection |
| *NF1* | Variant detection | | |

**Table S3: Antibody-oligonucleotide conjugate panel for single-cell multi-omics sequencing.** The panel consists of 17 targets each listed with their antibody ID and the antibody clone.

| **Protein target** | **Antibody ID** | **Clone** |
|---|---|---|
| CD2 | D0367 anti-human CD2 | TS1/8 |
| CD3 | D0034 anti-human CD3 | UCHT1 |
| CD7 | D0066 anti-human CD7 | CD7-6B7 |
| CD10 | D0062 anti-human CD10 | HI10a |
| CD11b | D0161 anti-human CD11b | ICRF44 |
| CD13 | D0364 anti-human CD13 | WM15 |
| CD14 | D0081 anti-human CD14 | M5E2 |
| CD19 | D0050 anti-human CD19 | HIB19 |
| CD22 | D0393 anti-human CD22 | S-HCL-1 |
| CD33 | D0052 anti-human CD33 | P67.6 |
| CD34 | D0054 anti-human CD34 | 581 |
| CD38 | D0389 anti-human CD38 | HIT2 |
| CD45RA | D0063 anti-human CD45RA | HI100 |
| CD56 | D0047 anti-human CD56 | 5.1H11 |
| CD123 | D0064 anti-human CD123 | 6H6 |
| HLA-DR | D0159 anti-human HLA-DR | L243 |
| CD117 | D1260 anti-human CD117 | A3C6E2 |

**Table S4: Genetic characteristics of patients determined by routine sequencing analyses at diagnosis.** Selected single cell-guided targets are highlighted in red. *Abbreviations: Alternate, Alt; chromosome, Chr; human genome variation society cDNA nomenclature, HGVSc; HGVS protein nomenclature, HGVSp; patient, Pt; reference, Ref; split apart, Sa; variant allele frequency, VAF.*

| Pt. | Cytogenetics | Gene | HGVSc/HGVSp | Chr | Position | Ref | Alt | Consequence | VAF |
|---|---|---|---|---|---|---|---|---|---|
| **1** | Normal | *DNMT3A* | NM_022552.5: c.2711C>T p.P904L | 2 | 25457176 | G | A | Missense | 0.45 |
| | | *IDH1* | NM_005896.4:c.394C>T p.R132C | 2 | 209113113 | G | A | Missense | 0.47 |
| | | *SRSF2* | NM_001195427.2: c.284C>A p.P95H | 17 | 74732959 | G | T | Missense | 0.46 |
| **2** | der(22) t(11;22) (q13;q13) | *ASXL1* | NM_015338.6:c.1900_1922del p.E635Rfs*15 | 20 | 31022415_31022437 | | | Frameshift | 0.47 |
| | | *EZH2* | NM_004456.4:c.1774_1777delACTT p.T592Vfs*82 | 7 | 148511128_148511131 | TAAG | - | Frameshift | 0.93 |
| | | *IDH1* | NM_005896.4:c.394C>T p.R132C | 2 | 209113113 | G | A | Missense | 0.42 |
| | | *TET2* | NM_001127208.3:c.3415delA p.I1139Lfs*13 | 4 | 106162501 | A | - | Frameshift | 0.47 |
| | | *TET2* | NM_001127208.3: c.4160A>G p.N1387S | 4 | 106183004 | A | G | Missense | 0.48 |
| **3** | Normal | *DNMT3A* | NM_022552.4:c.1555-2A>G p.? | 2 | 25467523 | T | C | N/A | 0.42 |
| | | *FLT3*(ITD) | NM_004119.3: c.1835_1836ins51 p.E611_F612ins17 | 13 | 28608223 | | | Insertion | 0.31 |
| | | *RUNX1* | NM_001754.4:c.908C>A p.S303* | 21 | 36171657 | G | T | Nonsense | 0.31 |
| **4** | Normal | *DNMT3A* | NM_022552.4:c.2120G>A p.G707D | 2 | 25463562 | C | T | Missense | 0.47 |
| | | *FLT3* | NM_004119.3:c.1775T>G p.V592G | 13 | 28608281 | A | C | Missense | 0.45 |
| | | *FLT3*(TKD) | NM_004119.2: c.2522A>T p.N841I | 13 | 28592623 | T | A | Missense | 0.03 |

| | | | | | | | | | |
|---|---|---|---|---|---|---|---|---|---|
| | | *IDH2* | NM_002168.2:c.419G>A p.R140Q | 15 | 90631934 | C | T | Missense | 0.49 |
| | | *PTPN11* | NM_002834.3: c.1505C>T p.S502L | 12 | 112926885 | C | T | Missense | 0.03 |
| **5** | Normal | *DNMT3A* | NM_022552.4:c.2644C>T p.R882C | 2 | 25457243 | G | A | Missense | 0.39 |
| | | *IDH2* | NM_002168.2:c.419G>A p.Arg140Gln | 15 | 90631934 | C | T | Missense | 0.25 |
| | | *SRSF2* | NM_003016.4:c.284C>A p.P95H | 17 | 74732959 | G | T | Missense | 0.40 |
| **6** | del(12p), add(16q) | *DNMT3A* | NM_022552.4: c.1917_1918del p.F640* | 2 | 25466790_ 25466791 | GA | - | Nonsense | 0.49 |
| | | *DNMT3A* | NM_022552.4: c.2185C>G p.R729G | 2 | 25463308 | G | C | Missense | 0.48 |
| | | *IDH1* | NM_005896.2:c.394C>G p.R132G | 2 | 209113113 | G | C | Missense | 0.48 |
| | | *SETBP1* | NM_015559.2: c.3233C>T p.T1078M | 18 | 42532538 | C | T | Missense | 0.47 |

**Figure S1: Deconvolution of clonality in first remission samples of relapsing patients.** Identified clones are listed with their genotypes and size. Only wildtype clones were identified in first remission samples of relapsing patients. Right: Identified wildtype clones are plotted against selected surface markers representing immature myelopoiesis, T-cell markers, B-cell markers, and mature myelopoiesis. Immunophenotypes are based on CLR-values of surface antigen expression, which can be found in Table S8.

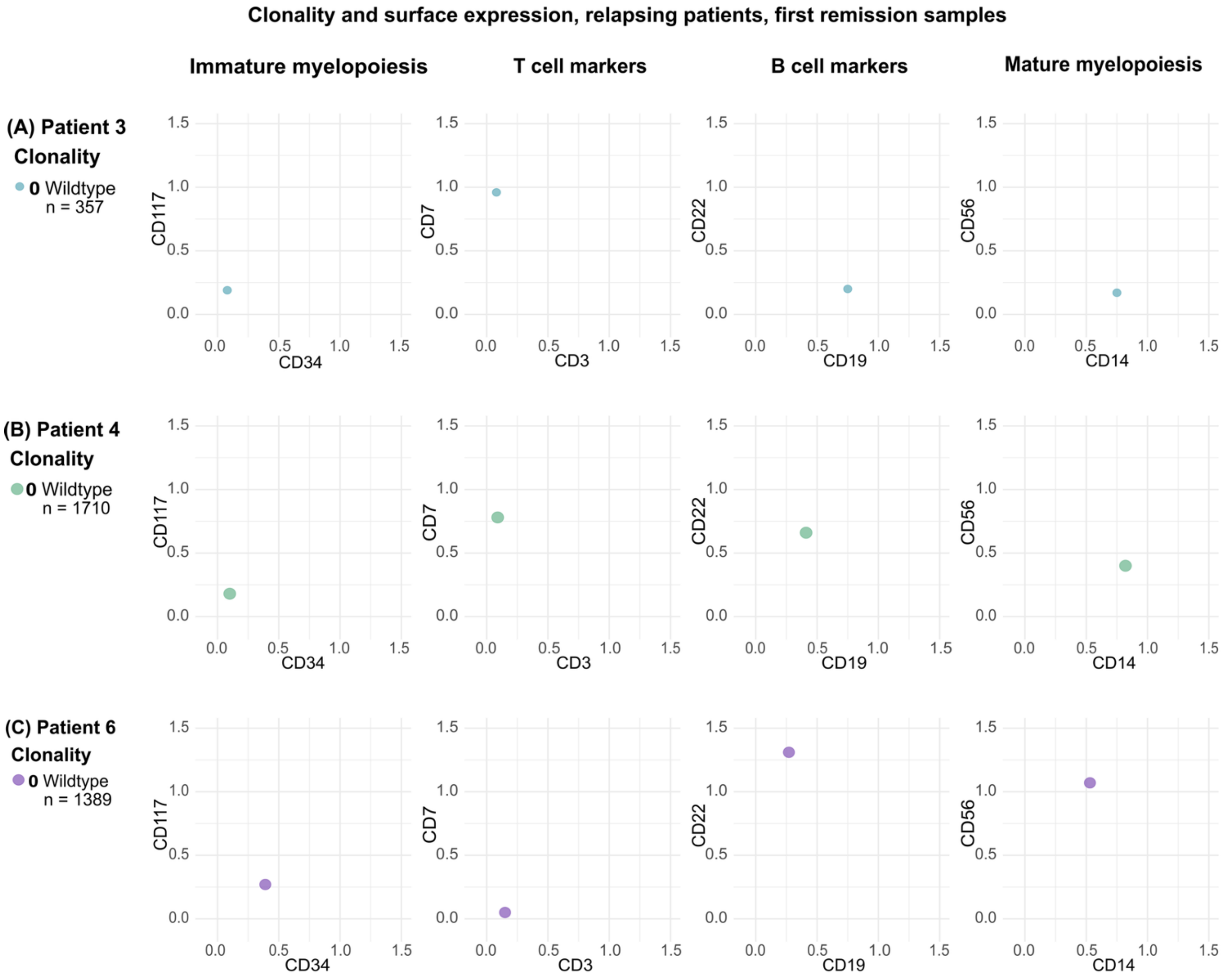

**Figure S2: Deconvolution of clonality in first remission samples of non-relapsing patients.** Left: Identified clones are listed with their genotypes and size. The clones are listed in evolutionary order. Right: Identified clones are plotted against selected surface markers representing immature myelopoiesis, T-cell markers, B-cell markers, and mature myelopoiesis. Immunophenotypes are based on CLR-values of surface antigen expression, which can be found in Table S8.

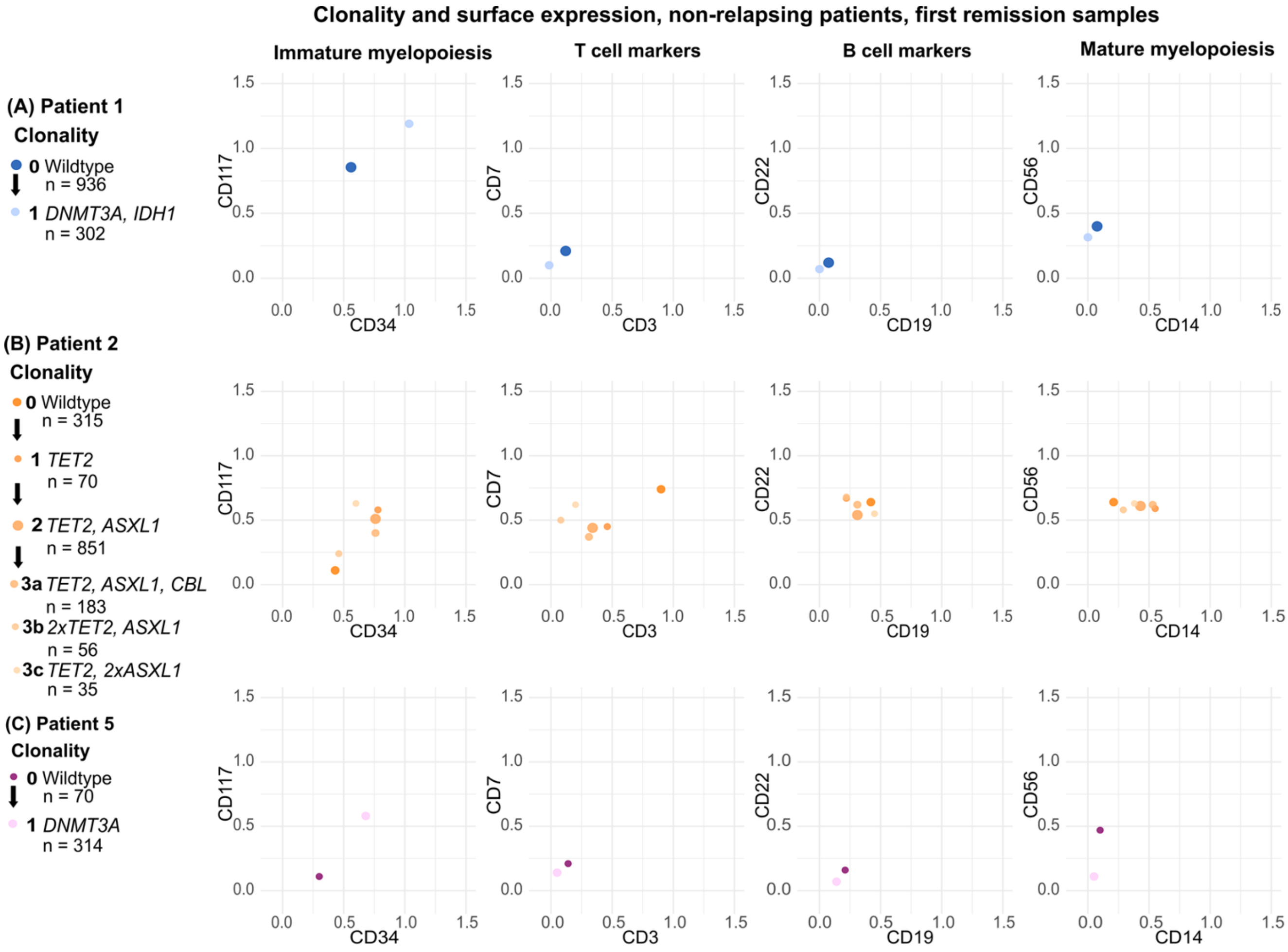

**Table S5: QC values of multiplexed single-cell multi-omics samples.** QC values were reported from the automated AML-MRD DNA+Protein Pipeline (Mission Bio) and measured before sequencing. *Abbreviations: Diagnosis, Dx; first remission, FR; multiplex, MP.*

| MP | #Cells | Panel uniformity | Mean reads/cell /amplicon | %Reads mapped to target | Mean reads/cell /antibody | DNA library input (ng/uL) | Protein library input (ng/uL) | Patient samples in multiplex (Patient no. and Dx or FR) |
|---|---|---|---|---|---|---|---|---|
| **A** | 8,806 | 93.46% | 76 | 90.72 | 514 | 18.6 | 8.77 | 1 Dx, 2 Dx, 3 Dx |
| **B** | 21,107 | 94.08% | 20 | 87.29 | 367 | 91.8 | 14.4 | 4 FR, 5 Dx, 6 FR |
| **C** | 10,943 | 91.28% | 56 | 80.25 | 581 | 1.24 | 3.38 | 1 FR, 3 FR |
| **D** | 10,266 | 78.82% | 40 | 87.99 | 1,096 | 9.81 | 13.7 | 2 FR |
| **E** | 23,013 | 95.02% | 12 | 62.73 | 244 | 1.24 | 4.04 | 6 Dx |
| **F** | 22,667 | 95.33% | 11 | 86.86 | 648 | 1.51 | 0.99 | 5 FR |
| **G** | 7,287 | 61.99% | 15 | 82.42 | 407 | 67.7 | 4.62 | 4 Dx |

**Table S6: Variants excluded using the five selection criteria (1-5) for target selection.**

| Selection criteria | Pitfall avoided | Targets excluded | | | | | |
|---|---|---|---|---|---|---|---|
| | | Patient 1 | Patient 2 | Patient 3 | Patient 4 | Patient 5 | Patient 6 |
| 1) The target is present in a large clone at diagnosis (target VAF≥10% across clones). | Subclonal variants | | | | | *PHF6* | |
| 2) The target is reduced to VAF<1% after the first treatment course. | Pre-leukemic variants | *IDH1*<br>*DNMT3A* | *ASXL1*<br>*TET2*<br>*TET2* | | | *DNMT3A* | |
| 3) The target is present in a clone with a distinct, immature immunophenotype, displaying aberrant characteristics. | Pre-leukemic variants | | *ASXL1*<br>*TET2*<br>*TET2* | | | | |
| 4) The target is not missing or lost in any clone at a more advanced stage (e.g., due to loss of heterozygozity, LOH). | Subclonal variants | | | | *FLT3* | *SRSF2*<br>*SMC1A*<br>*PHF6* | *DNMT3A* |
| 5) The target is not known to occasionally become negative at relapse. | Variants with low negative predictive values | | | *FLT3(ITD)* | *FLT3* | | |

*Abbreviations: Internal tandem duplication, ITD; loss of heterozygosity, LOH; variant allele frequency, VAF.*

**Table S7: Antigen expression of clones (>1) and wildtype cells (0) per diagnostic patient sample by single-cell multi-omics.** Values of antigen expression are centered log-transformed on the raw antibody counts.

| Pt. | Clone | Count | HLA-DR | CD7 | CD56 | CD45RA | CD38 | CD34 | CD33 | CD3 | CD22 | CD2 | CD19 | CD14 | CD13 | CD123 | CD11b | CD117 | CD10 |
|---|---|---|---|---|---|---|---|---|---|---|---|---|---|---|---|---|---|---|---|
| 1 | 0 | 34 | 0.67 | 1.3 | 0.76 | 1.02 | 0.62 | 0.53 | 0.54 | 1.16 | 0.84 | 0.88 | 0.87 | 0.08 | 0.86 | 0.68 | 0.15 | 0.06 | 0.07 |
| 1 | 1 | 857 | 1.03 | 0.02 | 0.1 | 0.96 | 0.81 | 1.02 | 0.66 | 0.04 | 0.07 | 0.01 | 0.02 | 0.02 | 0.75 | 0.91 | 0.04 | 1.29 | 0.04 |
| 1 | 2 | 254 | 1.01 | 0.03 | 0.08 | 0.94 | 0.82 | 1.01 | 0.26 | 0.02 | 0.07 | 0.01 | 0.01 | 0.03 | 0.73 | 0.92 | 0.04 | 1.31 | 0.02 |
| 1 | 3 | 2210 | 1.04 | 0.02 | 0.1 | 0.98 | 0.83 | 1.03 | 0.64 | 0.02 | 0.07 | 0.01 | 0.01 | 0.02 | 0.76 | 0.92 | 0.03 | 1.3 | 0.03 |
| 2 | 1 | 20 | 0.15 | 0.33 | 0.67 | 0.14 | 0.56 | 0.2 | 0.12 | 0.27 | 0.53 | 0.29 | 0.1 | 0.26 | 0.3 | 0.29 | 0.16 | 0.01 | 0.1 |
| 2 | 2 | 8 | 0.26 | 0.02 | 0.08 | -0.03 | 0.53 | 1.07 | 0.17 | -0.05 | 0.09 | 0.01 | -0.24 | -0.07 | 0.07 | 0.18 | 0.1 | 1.3 | -0.02 |
| 2 | 3 | 1928 | 1.26 | 0.04 | 1.39 | 0.02 | 0.53 | -0.01 | 0.89 | 0.02 | 0.07 | 0 | 0.02 | 0.01 | -0.01 | 0.39 | 0.05 | 0.53 | -0.01 |
| 3 | 0 | 60 | 0.27 | 0.14 | 0.15 | 0.46 | 0.3 | 0.7 | 0.24 | 0.11 | 0.25 | 0.15 | 0.17 | -0.06 | 0.32 | 0.13 | 0.07 | 0.62 | 0.11 |
| 3 | 1 | 2625 | 0.5 | 0.08 | 0.04 | 1.12 | 0.37 | 0.12 | 1.3 | 0.01 | 0.32 | 0.03 | 0.01 | 0 | 0.79 | 0.96 | 0.06 | 1.23 | 0 |
| 4 | 1 | 1614 | 0.1 | 0.01 | 1.33 | 0.01 | 0.49 | 0.13 | 1.01 | 0.01 | 0 | 0.01 | 0.01 | 0 | 0.04 | 0.3 | 0.01 | 1.11 | 0.02 |
| 4 | 2 | 499 | 0.08 | 0.01 | 1.33 | 0 | 0.48 | 0.1 | 0.98 | 0 | 0.01 | 0 | 0.01 | 0 | 0.04 | 0.32 | 0.01 | 1.15 | -0.01 |
| 4 | 3 | 1111 | 0.09 | 0.02 | 1.33 | 0.01 | 0.48 | 0.11 | 0.99 | 0 | 0 | 0.01 | 0.01 | 0.01 | 0.04 | 0.3 | 0.02 | 1.12 | 0.01 |
| 5 | 0 | 193 | 0.68 | 0.77 | 1.37 | 0.7 | 1.1 | 0.85 | 0.66 | 0.89 | 1.1 | 0.73 | 0.83 | 0.64 | 0.92 | 0.91 | 0.9 | 0.75 | 0.77 |
| 5 | 1 | 149 | 1.07 | 0.61 | 0.9 | 0.85 | 0.88 | 1.27 | 0.84 | 0.73 | 1.05 | 0.14 | 0.74 | 0.17 | 0.84 | 0.96 | 0.88 | 1.33 | 0.61 |
| 5 | 2 | 339 | 1.07 | 0.55 | 0.88 | 0.91 | 0.9 | 1.36 | 0.87 | 0.66 | 1.06 | 0.05 | 0.68 | 0.18 | 0.84 | 0.98 | 0.86 | 1.28 | 0.72 |
| 5 | 3 | 643 | 1.09 | 0.13 | 0.86 | 1.11 | 0.97 | 1.45 | 1.16 | 0.64 | 1.05 | 0.04 | 0.64 | 0.09 | 0.85 | 1.01 | 0.82 | 1.21 | 0.63 |
| 5 | 4 | 11142 | 1.12 | 0.14 | 0.85 | 1.12 | 0.96 | 1.47 | 1.16 | 0.67 | 1.03 | 0.03 | 0.63 | 0.11 | 0.85 | 1 | 0.82 | 1.23 | 0.59 |
| 5 | 5a | 4525 | 1.11 | 0.14 | 0.85 | 1.13 | 0.96 | 1.47 | 1.16 | 0.67 | 1.03 | 0.02 | 0.63 | 0.1 | 0.85 | 1 | 0.83 | 1.23 | 0.6 |
| 5 | 5b | 21 | 1.1 | 0.09 | 0.88 | 1.15 | 0.96 | 1.51 | 1.16 | 0.64 | 0.96 | -0.1 | 0.73 | 0.17 | 0.82 | 1 | 0.83 | 1.24 | 0.57 |
| 6 | 0 | 1683 | 0.13 | 1.41 | 0.05 | 0.59 | 0.36 | 0.59 | 0.19 | 0.05 | 0.02 | 0.06 | 0.06 | 0.01 | 0.02 | 0.2 | 0.02 | 0.72 | 0.01 |
| 6 | 1 | 8415 | 0.11 | 1.47 | 0.06 | 0.59 | 0.43 | 0.67 | 0.31 | 0.01 | 0.01 | 0.01 | 0.03 | 0 | 0.03 | 0.22 | 0.02 | 0.83 | 0.01 |
| 6 | 2 | 1899 | 0.14 | 1.46 | 0.06 | 0.61 | 0.43 | 0.66 | 0.36 | 0.02 | 0.01 | 0.03 | 0.03 | 0.01 | 0.03 | 0.22 | 0.04 | 0.86 | 0.01 |
| 6 | 3 | 8344 | 0.13 | 1.47 | 0.07 | 0.6 | 0.43 | 0.66 | 0.34 | 0.02 | 0.01 | 0.01 | 0.04 | 0.01 | 0.02 | 0.22 | 0.03 | 0.86 | 0.01 |
| 6 | 4 | 905 | 0.1 | 1.48 | 0.04 | 0.61 | 0.41 | 0.65 | 0.29 | 0.04 | 0.02 | 0.03 | 0.04 | 0.01 | 0.05 | 0.23 | 0.02 | 0.86 | 0 |

**Table S8: Antigen expression of clones (>1) and wildtype cells (0) per first remission patient sample by single-cell multi-omics.** Values of antigen expression are centered log-transformed on the raw antibody counts.

| Pt. | Clone | Count | HLA-DR | CD7 | CD56 | CD45RA | CD38 | CD34 | CD33 | CD3 | CD22 | CD2 | CD19 | CD14 | CD13 | CD123 | CD11b | CD117 | CD10 |
|---|---|---|---|---|---|---|---|---|---|---|---|---|---|---|---|---|---|---|---|
| 1 | 0 | 936 | 0.85 | 0.21 | 0.4 | 0.12 | 0.385 | 0.56 | 0.625 | 0.12 | 0.12 | 0.135 | 0.075 | 0.075 | 0.18 | 0.305 | 0.11 | 0.855 | 0.085 |
| 1 | 1 | 302 | 0.975 | 0.1 | 0.315 | 0.23 | 0.4 | 1.035 | 0.69 | -0.015 | 0.07 | 0 | 0 | 0 | 0.07 | 0.365 | 0.055 | 1.19 | 0.05 |
| 2 | 0 | 315 | 0.63 | 0.74 | 0.64 | 0.48 | 0.38 | 0.43 | 0.46 | 0.9 | 0.64 | 0.62 | 0.42 | 0.21 | 0.55 | 0.57 | 0.37 | 0.11 | 0.28 |
| 2 | 1 | 70 | 0.94 | 0.45 | 0.59 | 0.34 | 0.63 | 0.78 | 1.1 | 0.46 | 0.67 | 0.34 | 0.22 | 0.55 | 0.89 | 0.91 | 0.68 | 0.58 | 0.25 |
| 2 | 2 | 851 | 1.03 | 0.44 | 0.61 | 0.39 | 0.64 | 0.76 | 0.99 | 0.34 | 0.54 | 0.31 | 0.31 | 0.43 | 0.85 | 0.81 | 0.62 | 0.51 | 0.33 |
| 2 | 3 | 183 | 1.09 | 0.37 | 0.62 | 0.39 | 0.67 | 0.76 | 1.14 | 0.31 | 0.62 | 0.28 | 0.31 | 0.53 | 0.9 | 0.83 | 0.82 | 0.4 | 0.39 |
| 2 | 4 | 56 | 1.17 | 0.5 | 0.58 | 0.55 | 0.66 | 0.46 | 0.64 | 0.08 | 0.68 | 0.35 | 0.22 | 0.29 | 0.64 | 0.59 | 0.37 | 0.24 | 0.42 |
| 2 | 5 | 35 | 1.04 | 0.62 | 0.63 | 0.35 | 0.67 | 0.6 | 0.89 | 0.2 | 0.55 | 0.35 | 0.45 | 0.38 | 0.89 | 0.72 | 0.55 | 0.63 | 0.55 |
| 3 | 0 | 357 | 0.23 | 0.96 | 0.17 | 0.29 | 0.21 | 0.08 | 0.15 | 0.08 | 0.2 | 0.09 | 0.75 | 0.75 | 0.58 | 0.19 | 0.57 | 0.19 | 0.77 |
| 4 | 0 | 1710 | 0.24 | 0.78 | 0.4 | 0.48 | 0.35 | 0.1 | 0.21 | 0.09 | 0.66 | 0.2 | 0.41 | 0.82 | 0.28 | 0.16 | 0.41 | 0.18 | 0.7 |
| 5 | 0 | 70 | 0.25 | 0.21 | 0.47 | 0.33 | 0.22 | 0.3 | 0.03 | 0.14 | 0.16 | 0.12 | 0.21 | 0.1 | 0.19 | 0.11 | 0.17 | 0.11 | 0.16 |
| 5 | 1 | 314 | 0.62 | 0.14 | 0.11 | 0.3 | 0.13 | 0.68 | 0.12 | 0.05 | 0.07 | 0.01 | 0.14 | 0.05 | 0.12 | 0.16 | 0.14 | 0.58 | 0.19 |
| 6 | 0 | 1389 | 0.55 | 0.05 | 1.07 | 0.88 | 1.1 | 0.39 | 0.68 | 0.15 | 1.31 | 0.78 | 0.27 | 0.53 | 0.22 | 0.28 | 1.13 | 0.27 | 0.49 |

**Figure S3: CD34 expression (x-axis) vs CD117 expression (y-axis) of cells in the diagnostic samples of patients 4-6.** Cells are color-coded by their clonality. A) Patient 4: Clone 1 = *DNMT3A*; clone 2 = *DNMT3A*, *FLT3*; clone 3 = *DNMT3A*, *FLT3*, *IDH2*. B) Patient 5: Clone 1 = *DNMT3A*; clone 2 = *DNMT3A*, *SRSF2*; clone 3 = *DNMT3A*, *SRSF2*, *IDH2*; clone 4 = *DNMT3A*, *SRSF2*, *IDH2*, *SMC1A*, LOH(*SMC1A*); clone 5a = *DNMT3A*, *SRSF2*, *IDH2*, *SMC1A*, LOH(*SMC1A*), LOH(*SRSF2*); clone 5b = *DNMT3A*, *SRSF2*, *IDH2*, *SMC1A*, LOH(*SMC1A*), *PHF6*. C) Patient 6: Clone 1 = *IDH1*; clone 2 = *IDH1*, *DNMT3A*; clone 3 = *IDH1*, 2x*DNMT3A*; clone 4 = *IDH1*, 2x*DNMT3A,* LOH(*DNMT3A*).

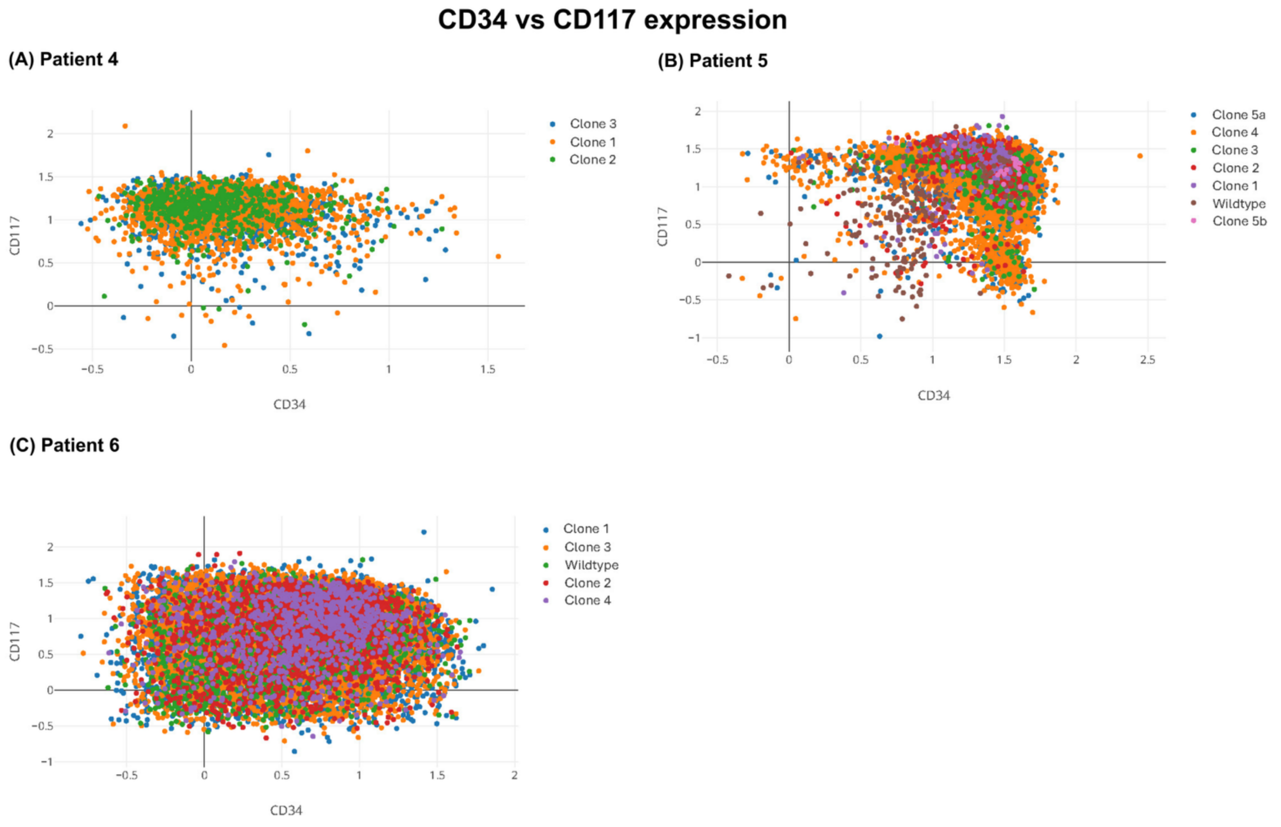

**Table S9: Sample availability for ddPCR analyses.** For each patient, the single-cell-guided MRD gene targets are listed along with the time from diagnosis until first remission assessment, pre-transplantation assessment, and transplantation. Additionally, the number of available PB samples and BM samples and the respective sampling intervals are listed. *Abbreviations: Bone marrow, BM; European LeukemiaNet, ELN; peripheral blood, PB; transplantation, Tx.*

| **Patient** | **1** | **2** | **3** | **4** | **5** | **6** |
|---|---|---|---|---|---|---|
| Relapse occurrence | No | No | Yes | Yes | No | Yes |
| Single cell-guided target | *SRSF2* | *EZH2/IDH2* | *RUNX1* | *DNMT3A/IDH2* | *IDH2* | *DNMT3A* |
| Post 1$^{st}$ cure | +35 | +48 | +47 | +40 | +61 | +30 |
| Pre Tx | +169 | +101 | +398 | - | +104 | +118 |
| Tx | +192 | +126 | +422 | - | +128 | +143 |
| Number of PB samples | 5 | 4/4 | 29 | 12/19 | 6 | 6 |
| Median interval (days) | 57 | 53/53 | 30 | 63/42.5 | 61 | 53 |
| Min. sampling interval | 35 | 48/48 | 6 | 36/14 | 43 | 27 |
| Max. sampling interval | 134 | 114/114 | 174 | 147/65 | 91 | 61 |
| Number of BM samples | 4 | 6/6 | 11 | 7/7 | 6 | 4 |
| Median interval (days) | 114 | 91/91 | 80 | 49.5/49.5 | 91 | 61 |
| Min. sampling interval | 35 | 48/48 | 8 | 36/36 | 44 | 30 |
| Max. sampling interval | 134 | 183/183 | 308 | 512/512 | 186 | 139 |

**Table S10: Descriptions of symbols indicating disease status by non-ddPCR methodologies.** For each sample assessed with ddPCR, a (+) or (-) indicates the MRD status as established by conventional non-ddPCR methodologies in the clinical practice.

| Symbol | Status | Description |
|---|---|---|
| Diamond | Disease | Immunologic: FCM ≥ 5% blasts<br>Morphologic: Blasts in PB and/or ≥ 5% blasts in BM smear and/or presence of blastoid foci. |
| Square | CR MRD(+) | 1. Complete morphologic remission and/or less than 5% blasts by FCM<br>2. FCM-MRD(+) in PB and/or BM, or<br>3. qPCR-MRD(+) in PB and/or BM, or<br>4. Abnormal metaphases and/or FISH(+) |
| Triangle | CR MRD(-) Partial Assessment | MRD(-) in all MRD technologies that were used (only a subset of available technologies). |
| Circle | CR MRD(-) Complete Assessment | 1. Complete morphologic remission and/or less than 5% blasts by FCM<br>2. MRD(-) in all available MRD technologies:<br>a. FCM-MRD(-) in PB and BM<br>b. qPCR-MRD(-) in PB and BM (if WT1 overexpression at diagnosis)<br>c. Normal metaphases and FISH(-) (if cytogenetically abnormal at diagnosis) |

*Abbreviations: Bone Marrow, BM; droplet digital PCR, ddPCR; flow cytometry, FCM; fluorescence in-situ hybridization, FISH; measurable residual disease, MRD; peripheral blood, PB; quantitative PCR, qPCR.*

**Table S11: Patient 1, table with VAFs quantified using targeted sequencing supporting Figure S4A.** The single cell-guided target for the patient was *SRSF2* p.P95H. Additional variants detected in the patient was *DNMT3A* p.P904L and *IDH1* p.R132C. The sensitivities for each target depended on the sample: *SRSF2* p.P95H ranged between 0.046-0.059%, *DNMT3A* p.P904L ranged between 0.18-0.21%, and *IDH1* p.R132C ranged between 0.19-0.21%.

| **Target: *SRSF2* p.P95H** | | | | | | | |
|---|---|---|---|---|---|---|---|
| **Days since diagnosis** | **Tissue** | **EC-NGS (VAF, %)** | **Morphology** | **MFC** | **WT1** | **Overall Non-NGS Status** | **Symbol** |
| 0 | BM | 45.8 | Disease | Disease | Disease | Disease | Diamond |
| 35 | BM | Negative | CR | CR MRD(+) | CR MRD(+) | CR MRD(+) | Square |
| 80 | BM | Negative | CR | CR MRD(+) | CR MRD(+) | CR MRD(+) | Square |
| 169 | BM | Negative | CR | CR MRD(+) | CR MRD(+) | CR MRD(+) | Square |
| 283 | BM | Negative | CR | CR MRD(+) | CR MRD(+) | CR MRD(+) | Square |
| **Additional variant: *DNMT3A* p.P904L** | | | | | | | |
| **Days since diagnosis** | **Tissue** | **EC-NGS (VAF, %)** | **Morphology** | **MFC** | **WT1** | **Overall Non-NGS Status** | **Symbol** |
| 0 | BM | 46.7 | Disease | Disease | Disease | Disease | Diamond |
| 35 | BM | 3.3 | CR | CR MRD(+) | CR MRD(+) | CR MRD(+) | Square |
| 80 | BM | 2.87 | CR | CR MRD(+) | CR MRD(+) | CR MRD(+) | Square |
| 169 | BM | 16.0 | CR | CR MRD(+) | CR MRD(+) | CR MRD(+) | Square |
| 283 | BM | Negative | CR | CR MRD(+) | CR MRD(+) | CR MRD(+) | Square |
| **Additional variant: *IDH1* p.R132C** | | | | | | | |
| **Days since diagnosis** | **Tissue** | **EC-NGS (VAF, %)** | **Morphology** | **MFC** | **WT1** | **Overall Non-NGS Status** | **Symbol** |
| 0 | BM | 45.9 | Disease | Disease | Disease | Disease | Diamond |
| 35 | BM | 2.8 | CR | CR MRD(+) | CR MRD(+) | CR MRD(+) | Square |
| 80 | BM | 2.5 | CR | CR MRD(+) | CR MRD(+) | CR MRD(+) | Square |
| 169 | BM | 15.2 | CR | CR MRD(+) | CR MRD(+) | CR MRD(+) | Square |
| 283 | BM | Negative | CR | CR MRD(+) | CR MRD(+) | CR MRD(+) | Square |

*Abbreviations: Bone Marrow, BM; complete remission, CR; error-corrected next-generation sequencing, EC-NGS; measurable residual disease, MRD; multiparameter flow cytometry, MFC; variant allele frequency, VAF.*

**Table S12: Patient 2, table with ddPCR quantified VAFs supporting Figure S4B.** The patient was followed with two targets, target 1: *EZH2* p.T592* (LoD = 0.0010934) and target 2: *IDH1* p.R132C (LoD = 0.0590341). In the case of *EZH2*, where the assay is extremely sensitive, the DNA input becomes the limiting factor, making RoT the defining criterion for the detection limit. Negative samples are highlighted in light red.

| **Target 1: *EZH2* p.T592* (LoD = 0.0010934)** | | | | | | | |
|---|---|---|---|---|---|---|---|
| **Days since diagnosis** | **Tissue** | **ddPCR VAF (%)** | **Morphology** | **MFC** | **Cytogenetics** | **Overall Non-ddPCR Status** | **Symbol** |
| 0 | PB | 81.99 | Disease | Disease | Disease | Disease | Diamond |
| 0 | BM | 91.52 | Disease | Disease | Disease | Disease | Diamond |
| 48 | PB | 0.04 | CR | CR MRD(+) | CR MRD(+) | CR MRD(+) | Square |
| 48 | BM | 0.07 | CR | CR MRD(+) | CR MRD(+) | CR MRD(+) | Square |
| 101 | PB | 0.01 | CR | CR | CR | CR MRD(-) Complete Assessment | Circle |
| 101 | BM | 0.06 | CR | CR | CR | CR MRD(-) Complete Assessment | Circle |
| 215 | PB | 0.00419820 | CR | CR | CR | CR MRD(-) Complete Assessment | Circle |
| 215 | BM | 0.00344845 | CR | CR | CR | CR MRD(-) Complete Assessment | Circle |
| 306 | BM | 0.00514133 | CR | CR | CR | CR MRD(-) Complete Assessment | Circle |
| 489 | BM | 0.00206224 | CR | CR | CR | CR MRD(-) Complete Assessment | Circle |
| **Target 2: *IDH1* p.R132C (LoD = 0.0590341)** | | | | | | | |
| **Days since diagnosis** | **Tissue** | **ddPCR VAF (%)** | **Morphology** | **MFC** | **Cytogenetics** | **Overall Non-ddPCR Status** | **Symbol** |
| 0 | PB | 35.03 | Disease | Disease | Disease | Disease | Diamond |
| 0 | BM | 42.13 | Disease | Disease | Disease | Disease | Diamond |
| 48 | PB | 0.05903405 | CR | CR MRD(+) | CR MRD(+) | CR MRD(+) | Square |
| 48 | BM | 0.05903405 | CR | CR MRD(+) | CR MRD(+) | CR MRD(+) | Square |
| 101 | PB | 0.05903405 | CR | CR | CR | CR MRD(-) Complete Assessment | Circle |
| 101 | BM | 0.05903405 | CR | CR | CR | CR MRD(-) Complete Assessment | Circle |
| 215 | PB | 0.05903405 | CR | CR | CR | CR MRD(-) Complete Assessment | Circle |
| 215 | BM | 0.05903405 | CR | CR | CR | CR MRD(-) Complete Assessment | Circle |
| 306 | BM | 0.05903405 | CR | CR | CR | CR MRD(-) Complete Assessment | Circle |
| 489 | BM | 0.05903405 | CR | CR | CR | CR MRD(-) Complete Assessment | Circle |

*Abbreviations: Bone Marrow, BM; complete remission, CR; droplet digital PCR, ddPCR; measurable residual disease, MRD; multiparameter flow cytometry, MFC; peripheral blood, PB; variant allele frequency, VAF.*

**Table S13: Patient 3, table with ddPCR quantified VAFs supporting Figure 3A.** The patient was followed with *RUNX1* p.S303* (LoD = 0.0096616). Negative samples are highlighted in light red. Relapses detected by ddPCR (ddPCR R) or Non-ddPCR (Non-ddPCR R) are marked with red text.

| Days since diagnosis | Tissue | ddPCR VAF (%) | Morphology | MFC | WT1 | Overall Non-ddPCR Status | Symbol |
|---|---|---|---|---|---|---|---|
| 0 | PB | 5 | Disease | Disease | Elevated | Disease | Diamond |
| 0 | BM | 28 | Disease | Disease | Elevated | Disease | Diamond |
| 47 | PB | 0.00960616 | CR | CR MRD(+) | Normal | CR MRD(+) | Square |
| 47 | BM | 0.01252874 | CR | CR MRD(+) | Normal | CR MRD(+) | Square |
| **ddPCR R (1)** 86 | PB | 0.02 | NA | NA | Normal | CR MRD(-) Partial Assessment | Triangle |
| 260 | PB | 0.02 | NA | NA | Normal | CR MRD(-) Partial Assessment | Triangle |
| 349 | PB | 0.50 | NA | NA | Normal | CR MRD(-) Partial Assessment | Triangle |
| **Non-ddPCR R (1)** 355 | PB | 0.60 | NA | CR MRD(-) | Elevated | CR MRD(+) | Square |
| **Non-ddPCR R (1)** 355 | BM | 2.20 | NA | CR MRD(-) | Elevated | CR MRD(+) | Square |
| 373 | PB | 1.91 | NA | NA | Elevated | CR MRD(+) | Square |
| 379 | PB | 3.54 | NA | NA | Normal | CR MRD(-) Partial Assessment | Triangle |
| 398 | PB | 1.82 | CR | CR MRD(-) | Normal | CR MRD(-) Complete Assessment | Circle |
| 398 | BM | 1.91 | CR | CR MRD(-) | Normal | CR MRD(-) Complete Assessment | Circle |
| 454 | PB | 0.00960616 | NA | NA | Normal | CR MRD(-) Partial Assessment | Triangle |
| 484 | PB | 0.00960616 | NA | NA | Normal | CR MRD(-) Partial Assessment | Triangle |
| 516 | PB | 0.00960616 | CR | CR MRD(-) | Normal | CR MRD(-) Complete Assessment | Circle |
| 516 | BM | 0.00960616 | CR | CR MRD(-) | Normal | CR MRD(-) Complete Assessment | Circle |
| **ddPCR R (2)** 558 | PB | 0.02 | NA | NA | Normal | CR MRD(-) Partial Assessment | Triangle |
| 587 | PB | 0.01 | CR | CR MRD(-) | Normal | CR MRD(-) Complete Assessment | Circle |
| 587 | BM | 0.05 | CR | CR MRD(-) | Normal | CR MRD(-) Complete Assessment | Circle |
| 616 | PB | 0.10 | NA | NA | Normal | CR MRD(-) Partial Assessment | Triangle |
| 645 | PB | 0.20 | NA | NA | Normal | CR MRD(-) Partial Assessment | Triangle |
| **Non-ddPCR R (2)** 673 | PB | 0.48 | CR | CR MRD(-) | Elevated | CR MRD(+) | Square |
| **Non-ddPCR R (2)** 673 | BM | 0.72 | CR | CR MRD(-) | Elevated | CR MRD(+) | Square |
| 747 | PB | 7.18 | CR | NA | Elevated | CR MRD(+) | Square |
| 747 | BM | 9.54 | CR | NA | Elevated | CR MRD(+) | Square |
| 755 | PB | 1.93 | CR | CR MRD(-) | Elevated | CR MRD(+) | Square |
| 755 | BM | 8.14 | CR | CR MRD(-) | Elevated | CR MRD(+) | Square |

| | | | | | | | |
|---|---|---|---|---|---|---|---|
| 785 | PB | 1.82 | NA | NA | Elevated | CR MRD(+) | Square |
| 817 | PB | 2.74 | NA | NA | Elevated | CR MRD(+) | Square |
| 845 | PB | 1.83 | NA | NA | Elevated | CR MRD(+) | Square |
| 909 | PB | 2.96 | NA | NA | Elevated | CR MRD(+) | Square |
| 931 | PB | 2.50 | NA | NA | Normal | CR MRD(-) Partial Assessment | Triangle |
| 943 | PB | 4.60 | NA | NA | Elevated | CR MRD(+) | Square |
| 964 | PB | 3.38 | NA | NA | Normal | CR MRD(-) Partial Assessment | Triangle |
| 1013 | PB | 6.28 | CR | NA | Elevated | CR MRD(+) | Square |
| 1013 | BM | 26.40 | CR | NA | Elevated | CR MRD(+) | Square |
| 1076 | PB | 3.77 | NA | NA | Elevated | CR MRD(+) | Square |
| 1128 | PB | 5.67 | Disease | NA | Elevated | Disease | Diamond |
| 1128 | BM | 40.50 | Disease | NA | Elevated | Disease | Diamond |

*Abbreviations: Bone Marrow, BM; complete remission, CR; droplet digital PCR, ddPCR; measurable residual disease, MRD; multiparameter flow cytometry, MFC; not available, NA; peripheral blood, PB; variant allele frequency, VAF.*

**Table S14: Patient 4, table with ddPCR quantified VAFs supporting Figure 3B.** The patient was followed with two targets, Target 1: *DNMT3A* p.G707D (LoD = 0.0390657) and Target 2: *IDH2* p.R140Q (LoD = 0.06). Relapsed detected by ddPCR (ddPCR R) or Non-ddPCR (Non-ddPCR R) are marked with red text.

| **Target 1: *DNMT3A* p.G707D (LoD = 0.0390657)** | | | | | | | |
|---|---|---|---|---|---|---|---|
| **Days since diagnosis** | **Tissue** | **ddPCR VAF (%)** | **Morphology** | **MFC** | **WT1** | **Overall Non-ddPCR Status** | **Symbol** |
| 0 | PB | 47.03 | Disease | Disease | Elevated | Disease | Diamond |
| 0 | BM | 48.26 | Disease | Disease | Elevated | Disease | Diamond |
| 40 | PB | 0.14 | CR | CR | Normal | CR MRD(-) Complete Assessment | Circle |
| 40 | BM | 0.25 | CR | CR | Normal | CR MRD(-) Complete Assessment | Circle |
| 76 | PB | 0.33 | CR | CR | Normal | CR MRD(-) Complete Assessment | Circle |
| 76 | BM | 0.57 | CR | CR | Normal | CR MRD(-) Complete Assessment | Circle |
| 130 | PB | 0.22 | NA | NA | Normal | CR MRD(-) Partial Assessment | Triangle |
| 130 | BM | 0.38 | NA | NA | Normal | CR MRD(-) Partial Assessment | Triangle |
| 175 | PB | 0.24 | NA | NA | Normal | CR MRD(-) Partial Assessment | Triangle |
| 175 | BM | 0.19 | NA | NA | Normal | CR MRD(-) Partial Assessment | Triangle |
| 293 | PB | 0.96 | CR | CR | Normal | CR MRD(-) Complete Assessment | Circle |

| | | | | | | | |
|---|---|---|---|---|---|---|---|
| 293 | BM | 1.32 | CR | CR | Normal | CR MRD(-) Complete Assessment | Circle |
| 355 | PB | 1.70 | NA | NA | Normal | CR MRD(-) Partial Assessment | Triangle |
| **ddPCR R** 467 | PB | 2.28 | NA | NA | Normal | CR MRD(-) Partial Assessment | Triangle |
| 614 | PB | 5.06 | NA | NA | Normal | CR MRD(-) Partial Assessment | Triangle |
| 677 | PB | 12.67 | NA | NA | Normal | CR MRD(-) Partial Assessment | Triangle |
| 740 | PB | 23.64 | NA | NA | Normal | CR MRD(-) Partial Assessment | Triangle |
| **Non-ddPCR R** 805 | PB | 13.51 | CR | CR | Elevated | CR MRD(+) | Square |
| **Non-ddPCR R** 805 | BM | 31.11 | CR | CR | Elevated | CR MRD(+) | Square |
| **Target 2: *IDH2* p.R140Q (LoD = 0.06)** | | | | | | | |
| **Days since diagnosis** | **Tissue** | **ddPCR VAF (%)** | **Morphology** | **MFC** | **WT1** | **Overall Non-ddPCR Status** | **Symbol** |
| 0 | PB | 47.40 | Disease | Disease | Elevated | Disease | Diamond |
| 0 | BM | 49.40 | Disease | Disease | Elevated | Disease | Diamond |
| 40 | PB | 0.24 | CR | CR MRD (-) | Normal | CR MRD(-) Complete Assessment | Circle |
| 40 | BM | 0.21 | CR | CR MRD (-) | Normal | CR MRD(-) Complete Assessment | Circle |
| 76 | PB | 0.49 | CR | CR MRD (-) | Normal | CR MRD(-) Complete Assessment | Circle |
| 76 | BM | 0.53 | CR | CR MRD (-) | Normal | CR MRD(-) Complete Assessment | Circle |
| 90 | PB | 0.41 | NA | NA | Normal | CR MRD(-) Partial Assessment | Triangle |
| 130 | PB | 0.36 | NA | NA | Normal | CR MRD(-) Partial Assessment | Triangle |
| 130 | BM | 0.43 | NA | NA | Normal | CR MRD(-) Partial Assessment | Triangle |
| 175 | PB | 0.33 | NA | CR MRD (-) | Normal | CR MRD(-) Partial Assessment | Triangle |
| 175 | BM | 0.20 | NA | CR MRD (-) | Normal | CR MRD(-) Partial Assessment | Triangle |
| 222 | PB | 0.46 | NA | NA | Normal | CR MRD(-) Partial Assessment | Triangle |
| 257 | PB | 1.13 | NA | NA | Normal | CR MRD(-) Partial Assessment | Triangle |
| 293 | PB | 0.96 | CR | CR MRD (-) | Normal | CR MRD(-) Complete Assessment | Circle |
| 293 | BM | 1.35 | CR | CR MRD (-) | Normal | CR MRD(-) Complete Assessment | Circle |
| 355 | PB | 1.63 | NA | NA | Normal | CR MRD(-) Partial Assessment | Triangle |
| 376 | PB | 1.91 | NA | NA | Normal | CR MRD(-) Partial Assessment | Triangle |
| 411 | PB | 1.93 | NA | NA | Normal | CR MRD(-) Partial Assessment | Triangle |
| **ddPCR R** 467 | PB | 2.57 | NA | NA | Normal | CR MRD(-) Partial Assessment | Triangle |
| 502 | PB | 2.64 | NA | NA | Normal | CR MRD(-) Partial Assessment | Triangle |
| 558 | PB | 3.43 | NA | NA | Normal | CR MRD(-) Partial Assessment | Triangle |
| 614 | PB | 5.07 | NA | NA | Normal | CR MRD(-) Partial Assessment | Triangle |

| | | | | | | | |
|---|---|---|---|---|---|---|---|
| 677 | PB | 12.67 | NA | NA | Normal | CR MRD(-) Partial Assessment | Triangle |
| 740 | PB | 24.19 | NA | NA | Normal | CR MRD(-) Partial Assessment | Triangle |
| **Non-ddPCR R** 805 | PB | 13.74 | CR | CR MRD (+) | Elevated | CR MRD(+) | Square |
| **Non-ddPCR R** 805 | BM | 30.96 | CR | CR MRD (+) | Elevated | CR MRD(+) | Square |

*Abbreviations: Bone Marrow, BM; complete remission, CR; droplet digital PCR, ddPCR; measurable residual disease, MRD; multiparameter flow cytometry, MFC; not available, NA; peripheral blood, PB; variant allele frequency, VAF.*

**Table S15: Patient 5, table with ddPCR quantified VAFs supporting Figure S4C.** The patient was followed with *IDH2* p.R140Q (LoD = 0.06). Negative samples are highlighted in light red. Relapsed detected by ddPCR (ddPCR R) or Non-ddPCR (Non-ddPCR R) are marked with red text.

| Days since diagnosis | Tissue | ddPCR VAF (%) | Morphology | MFC | Cytogenetics | Overall Non-ddPCR Status | Symbol |
|---|---|---|---|---|---|---|---|
| 0 | PB | 0.50 | Disease | Disease | FISH disease* | Disease | Diamond |
| 0 | BM | 25.09 | Disease | Disease | FISH disease* | Disease | Diamond |
| 61 | PB | 0.06 | CR | CR MRD(-) | NA | CR MRD(-) Complete Assessment | Circle |
| 61 | BM | 0.06 | CR | CR MRD(-) | NA | CR MRD(-) Complete Assessment | Circle |
| 104 | PB | 0.06 | CR | CR MRD(-) | CR | CR MRD(-) Complete Assessment | Circle |
| 105 | BM | 0.06 | CR | CR MRD(-) | CR | CR MRD(-) Complete Assessment | Circle |
| 168 | PB | 0.06 | NA | NA | NA | CR MRD(-) Partial Assessment | Triangle |
| 211 | PB | 0.06 | CR | CR MRD(-) | CR | CR MRD(-) Complete Assessment | Circle |
| 211 | BM | 0.07 | CR | CR MRD(-) | CR | CR MRD(-) Complete Assessment | Circle |
| 302 | PB | 0.06 | CR | CR MRD(-) | MRD** | CR MRD(+) | Square |
| 302 | BM | 0.06 | CR | CR MRD(-) | MRD** | CR MRD(+) | Square |
| 491 | BM | 0.07 | CR | CR MRD(-) | CR | CR MRD(-) Complete Assessment | Circle |

*The standard chromosome analysis has not detected clonal changes. The 72-hour interphase nucleus FISH analysis has detected 6% trisomy 21. However, the FISH detected chromosomal abnormality is not considered leukemia-driving due to its low prevalence and association with intermediate risk. Thus, the criteria for a 'Complete Assessment' are met without the need for a FISH analysis.
**The standard chromosome analysis has detected x, 47,XY+mar[3]/46,XY[22]. The identified marker chromosome is newly emerged and prognostically non-specific; therefore, it is not interpreted as definite AML disease.

*Abbreviations: Bone Marrow, BM; complete remission, CR; droplet digital PCR, ddPCR; fluorescence in-situ hybridization, FISH; measurable residual disease, MRD; multiparameter flow cytometry, MFC; not available, NA; peripheral blood, PB; variant allele frequency, VAF.*

**Table S16: Patient 6, table with ddPCR quantified VAFs supporting Figure 3C.** The patient was followed with DNMT3A p.R729G (LoD = 0.00242). Relapsed detected by ddPCR (ddPCR R) or Non-ddPCR (Non-ddPCR R) are marked with red text.

| Days since diagnosis | Tissue | ddPCR VAF (%) | Morphology | MFC | Cytogenet-ics | Overall Non-ddPCR Status | Symbol |
|---|---|---|---|---|---|---|---|
| 0 | PB | 46.70 | Disease | Disease | Disease | Disease | Diamond |
| 0 | BM | 48.09 | Disease | Disease | NA | Disease | Diamond |
| 30 | PB | 0.0869 | CR | CR MRD(+) | CR | CR MRD(+) | Square |
| 30 | BM | 0.0141 | CR | CR MRD(+) | CR | CR MRD(+) | Square |
| 91 | PB | 0.0156 | NA | CR MRD(-) | CR | CR MRD(-) Complete Assessment | Circle |
| 91 | BM | 0.0088 | NA | CR MRD(-) | CR | CR MRD(-) Complete Assessment | Circle |
| 118 | PB | 0.0029 | NA | CR MRD(-) | CR | CR MRD(-) Complete Assessment | Circle |
| 171 | PB | 0.0135 | NA | NA | NA | CR MRD(-) Partial Assessment | Triangle |
| **ddPCR and non-ddPCR R** 230 | PB | 4.52 | Disease | Disease | Disease | Disease | Diamond |
| **ddPCR and non-ddPCR R** 230 | BM | 44.92 | Disease | Disease | Disease | Disease | Diamond |

*Abbreviations: Bone Marrow, BM; complete remission, CR; droplet digital PCR, ddPCR; measurable residual disease, MRD; multiparameter flow cytometry, MFC; not available, NA; peripheral blood, PB; variant allele frequency, VAF.*

**Figure S4: Highly sensitive ddPCR and EC-NGS for disease surveillance targeting single cell-guided MRD markers for non-relapsing AML.** Three non-relapsing AML patients lacking validated MRD markers were monitored by quantification of VAFs using (A) EC-NGS for *SRSF2* p.P95H, *DNMT3A* p.P904L, and *IDH1* p.R132C, (B) ddPCR for *EZH2* p.T592* and *IDH1* p.R132C, and (C) ddPCR for *IDH2* p.R140Q. PB and BM assessments are depicted using dotted and solid lines, respectively. Each data point is formatted according to the conventionally determined MRD status as defined in clinical practice, adhering to the ELN guidelines, Table S10. CR MRD(-) Partial Assessment is defined as MRD(-) in all MRD technologies that were used when only a subset of available technologies were analyzed. The target LoD thresholds are represented as horizontal lines. Negative samples (ddPCR VAF ≤ LoD; variant not significantly called using EC-NGS) are non-filled, whereas positive samples are color-filled (ddPCR VAF ≥ LoD; variant significantly called using EC-NGS). *Abbreviations: Bone marrow, BM; liposomal cytarabine and daunorubicin, CPX351; complete remission, CR; daunorubicin and cytarabine, DA; digital droplet PCR, ddPCR; error-corrected next-generation sequencing, EC-NGS; European LeukemiaNet, ELN; Fludarabine, arabinofuranosyl cytidine, granulocyte colony-stimulating factor, and idarubicin, FLAG-Ida; intravenous fludarabine, IV flu; limit of detection, LoD; midostaurin, Mido; measurable residual disease, MRD; non-myeloablative stem cell transplantation, NMA SCT; peripheral blood, PB; variant allele frequency, VAF.*

### (A) Patient 1

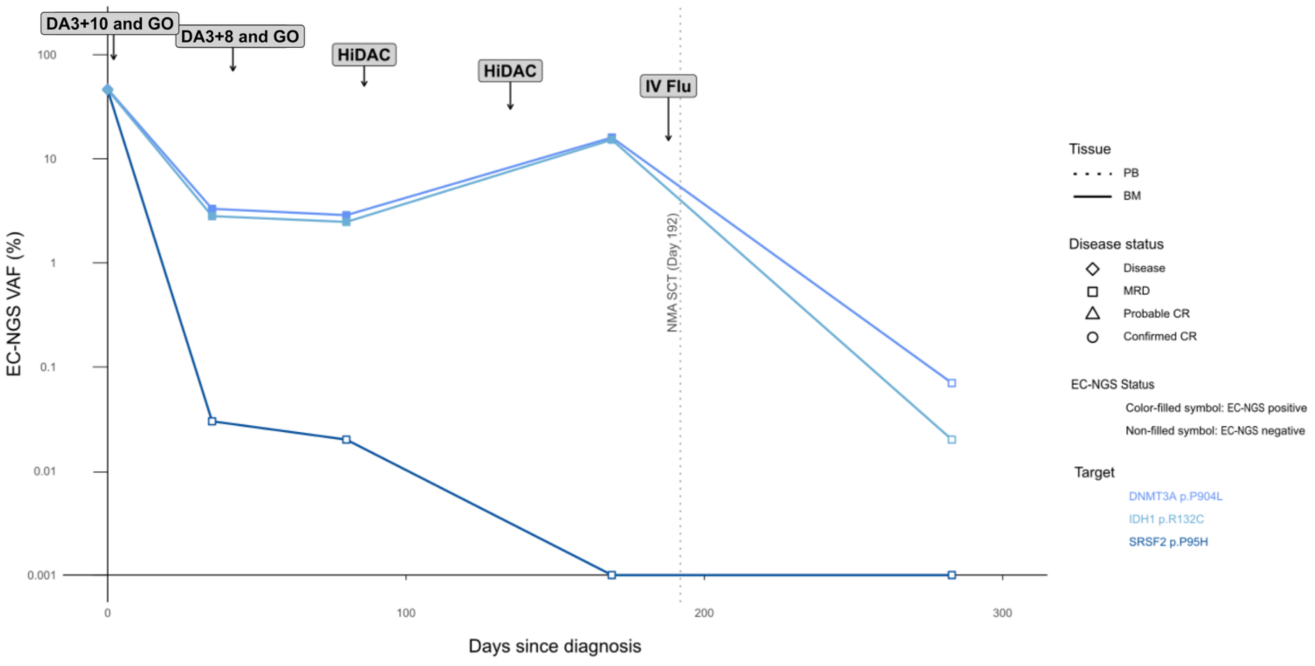

## (B) Patient 2

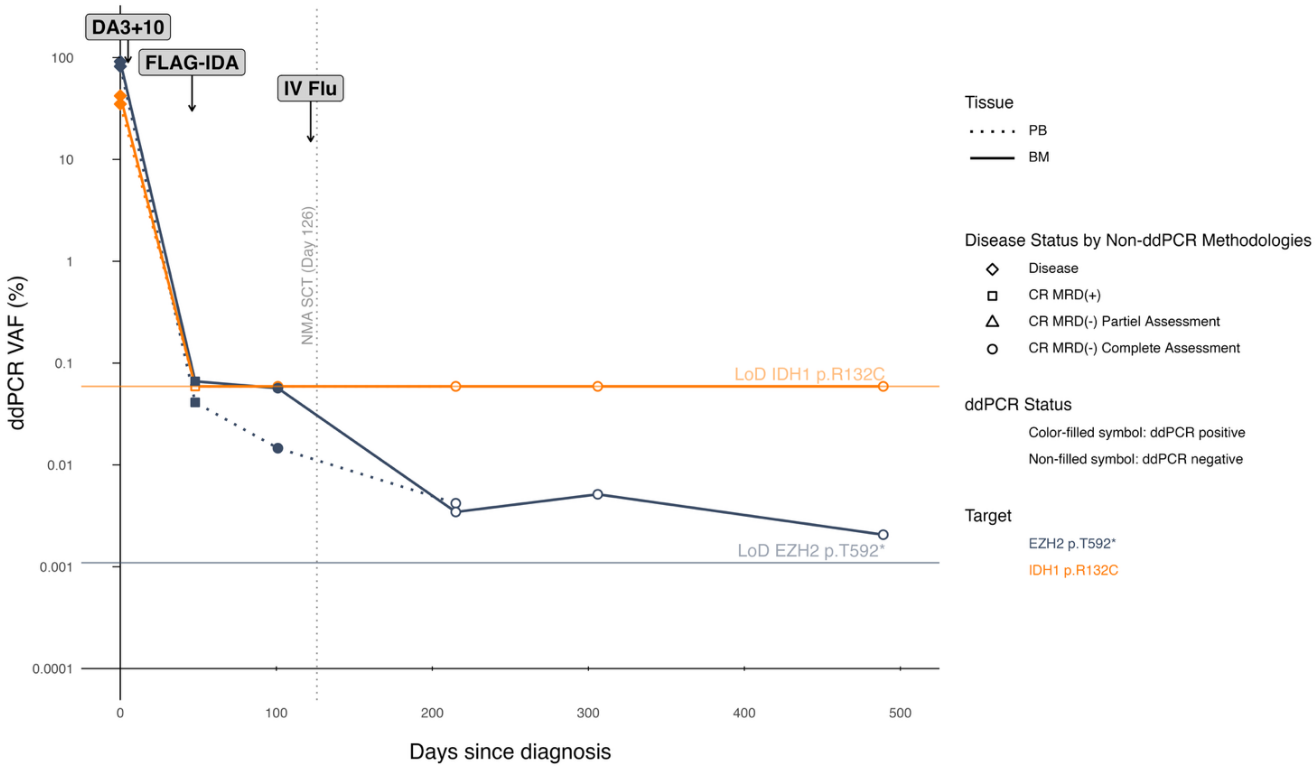


## (C) Patient 5

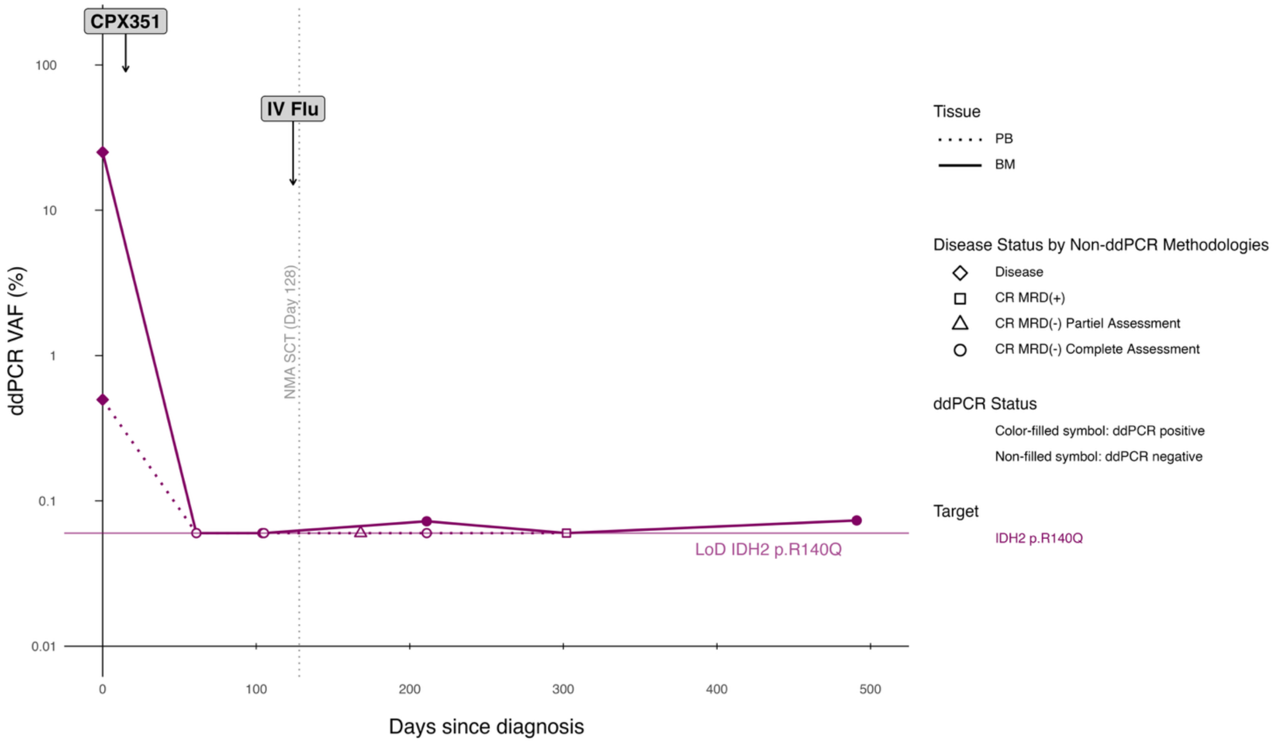

Supplement: Supplementary file 1 [file cancers-18-00787-s001.zip › Supplementary_270226.pdf]
